# Comprehensive analyses of m6A regulators and interactive coding and non-coding RNAs across 32 cancer types

**DOI:** 10.1186/s12943-021-01362-2

**Published:** 2021-04-13

**Authors:** Sipeng Shen, Ruyang Zhang, Yue Jiang, Yi Li, Lijuan Lin, Zhonghua Liu, Yang Zhao, Hongbing Shen, Zhibin Hu, Yongyue Wei, Feng Chen

**Affiliations:** 1 State Key Laboratory of Reproductive Medicine, Nanjing Medical University, Nanjing, 211166 China; 2 Jiangsu Key Lab of Cancer Biomarkers, Prevention and Treatment, Jiangsu Collaborative Innovation Center for Cancer Personalized Medicine, Nanjing Medical University, SPH Building Room 418, 101 Longmian Avenue, Nanjing, 211166 Jiangsu China; 3 Department of Biostatistics, Center for Global Health, School of Public Health, Nanjing Medical University, Nanjing, 211166 China; 4 China International Cooperation Center of Environment and Human Health, Nanjing Medical University, Nanjing, 211166 Jiangsu China; 5 Key Laboratory of Biomedical Big Data of Nanjing Medical University, Nanjing, 211166 China; 6 Department of Biostatistics, University of Michigan, Ann Arbor, MI 48109 USA; 7 Department of Statistics and Actuarial Science, University of Hong Kong, Hong Kong, SAR China; 8 Department of Epidemiology, Center for Global Health, School of Public Health, Nanjing Medical University, Nanjing, 211166 China

**Keywords:** N6-Methyladenosine, Pan-cancer, Survival outcome, Multi-omics

## Abstract

N6-Methyladenosine (m6A) is an RNA modification that interacts with numerous coding and non-coding RNAs and plays important roles in the development of cancers. Nonetheless, the clinical impacts of m6A interactive genes on these cancers largely remain unclear since most studies focus only on a single cancer type. We comprehensively evaluated m6A modification patterns, including 23 m6A regulators and 83 interactive coding and non-coding RNAs among 9,804 pan-cancer samples. We used clustering analysis to identify m6A subtypes and constructed the m6A signature based on an unsupervised approach. We used the signatures to identify potential m6A modification targets across the genome. The prognostic value of one target was further validated in 3,444 samples from six external datasets. We developed three distinct m6A modification subtypes with different tumor microenvironment cell infiltration degrees: immunological, intermediate, and tumor proliferative. They were significantly associated with overall survival in 24 of 27 cancer types. Our constructed individual-level m6A signature was associated with survival, tumor mutation burden, and classical pathways. With the signature, we identified 114 novel genes as potential m6A targets. The gene shared most commonly between cancer types, *BCL9L*, is an oncogene and interacts with m6A patterns in the Wnt signaling pathway. In conclusion, m6A regulators and their interactive genes impact the outcome of various cancers. Evaluating the m6A subtype and the signature of individual tumors may inform the design of adjuvant treatments.

**Supplementary Information:**

The online version contains supplementary material available at 10.1186/s12943-021-01362-2.

## Background

N6-Methyladenosine (m6A) is a eukaryotic mRNA modification that modulates gene expression [1, 2] and alters the fate of modified RNA molecules by changing mRNA stability, splicing, transport, localization, translation, microRNA (miRNA) processing, and RNA-protein interactions [3–5]. Emerging evidence suggests that m6A modifications are associated with tumor proliferation, differentiation, tumorigenesis, invasion, and metastasis [6, 7] and play important roles in cancer development [8, 9]. What’s more, m6A modifications are also regulated by numerous protein-coding genes [10–12] and non-coding RNAs (e.g., miRNAs, lncRNAs) interact by controlling cleavage, localization, transport, stability, and degradation and by influencing biological processes such as proliferation, infiltration, and metastasis of tumor cells [13–15]. However, few studies have comprehensively evaluated m6A interactive genes for both coding and non-coding RNAs, which contribute a lot to cancers. In this study, we investigated m6A regulators along with their interactive coding and non-coding RNAs in a pan-cancer setting.

## Results and discussions

### Landscape of m6A patterns in TCGA pan-cancer

**Fig. 1:**
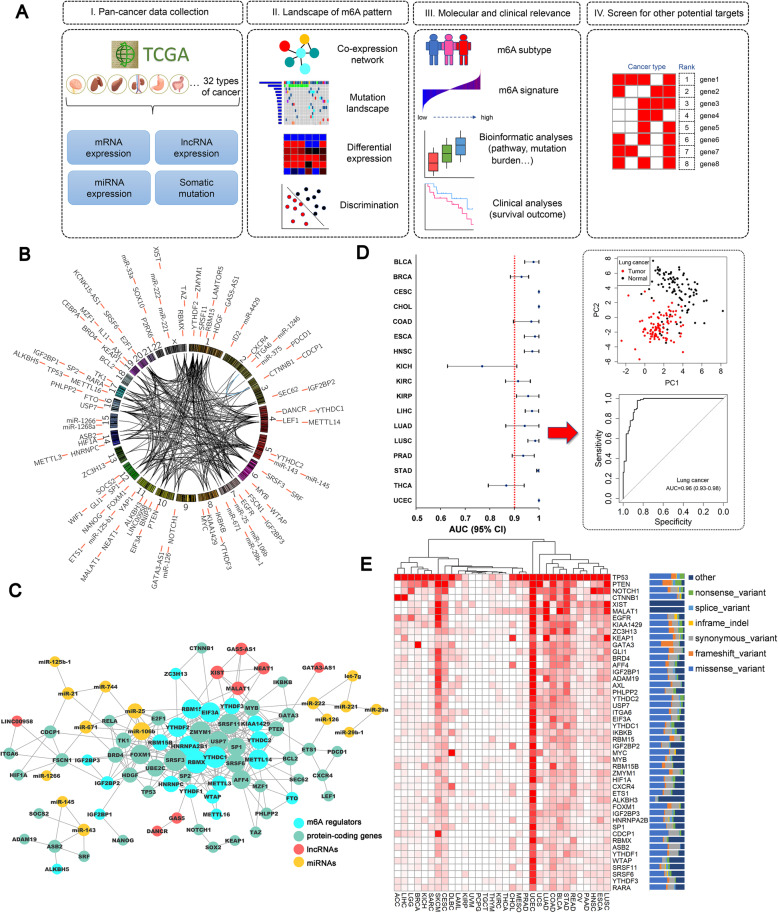
**a** Study workflow. **b** Circos plot of the selected genes on the chromosome. **c** Gene co-expression network of the m6A-related genes. The gene pairs with Pearson *r* > 0.3 are considered to have co-expression correlation. **d** Discrimination analyses of the tumor and adjacent normal tissues in pan-cancer (cancer types with ≥5 tumor-normal pairs included) based on the m6A gene panel. In the principal components plot of the m6A gene panel in lung cancer tumor/normal tissues, the area under the curve (AUC) of distinguishing between tumor and normal tissues is 0.96 (95% CI: 0.93–0.98). **e** Heatmap of the somatic mutation frequency of the genes across pan-cancer

To characterize m6A patterns and screen for potential targets, we developed a four-step computational framework among 9804 pan-cancer samples in The Cancer Genome Atlas (TCGA) (Additional file: Methods, Figure 1A). A total of 23 m6A regulators, 56 m6A interactive protein-coding genes, 10 lncRNAs, and 17 miRNAs were included in this study after quality control (Table S1). The 106 genes had close co-expression relations (Pearson *r* > 0.3) (Fig. 1b). In the co-expression regulation network, most of the m6A regulators and some of the protein-coding genes were hub genes that interacted with other genes (Fig. 1c).

In the differential expression comparisons of tumor-adjacent normal tissues across different cancer types, many genes had higher expression levels in tumor tissues while some protein-coding genes showed the opposite relationship (Figure S1). These genes included *ASB2, P2RX6, AXL, ID2,* and *SOCS2*. Four miRNAs including *miR-143, miR-29a, miR-125b-1*, and *miR-145* showed lower expression in tumor tissues. All of the lncRNAs had higher expression in tumor tissues. Using the first two principal components (PCs) from tumor and normal tissues, we found that the m6A patterns had good differential diagnostic value (Figure S2). The Area Under Curve (AUC) passed 90% in most cancers (Fig. 1d, S3).

The somatic mutation status of these genes is shown in Fig. 1e. The top genes with the highest average mutated frequency were *TP53* (31.4%)*, PTEN* (5.7%)*, NOTCH1* (3.6%)*, CTNNB1* (3.6%)*, XIST* (3.4%), and *MALAT1* (3.4%). We also observed a relatively high mutation frequency in uterine corpus endometrial carcinoma (UCEC) (7.6%).

### Constructing m6A subtypes and signature in pan-cancer

**Fig. 2:**
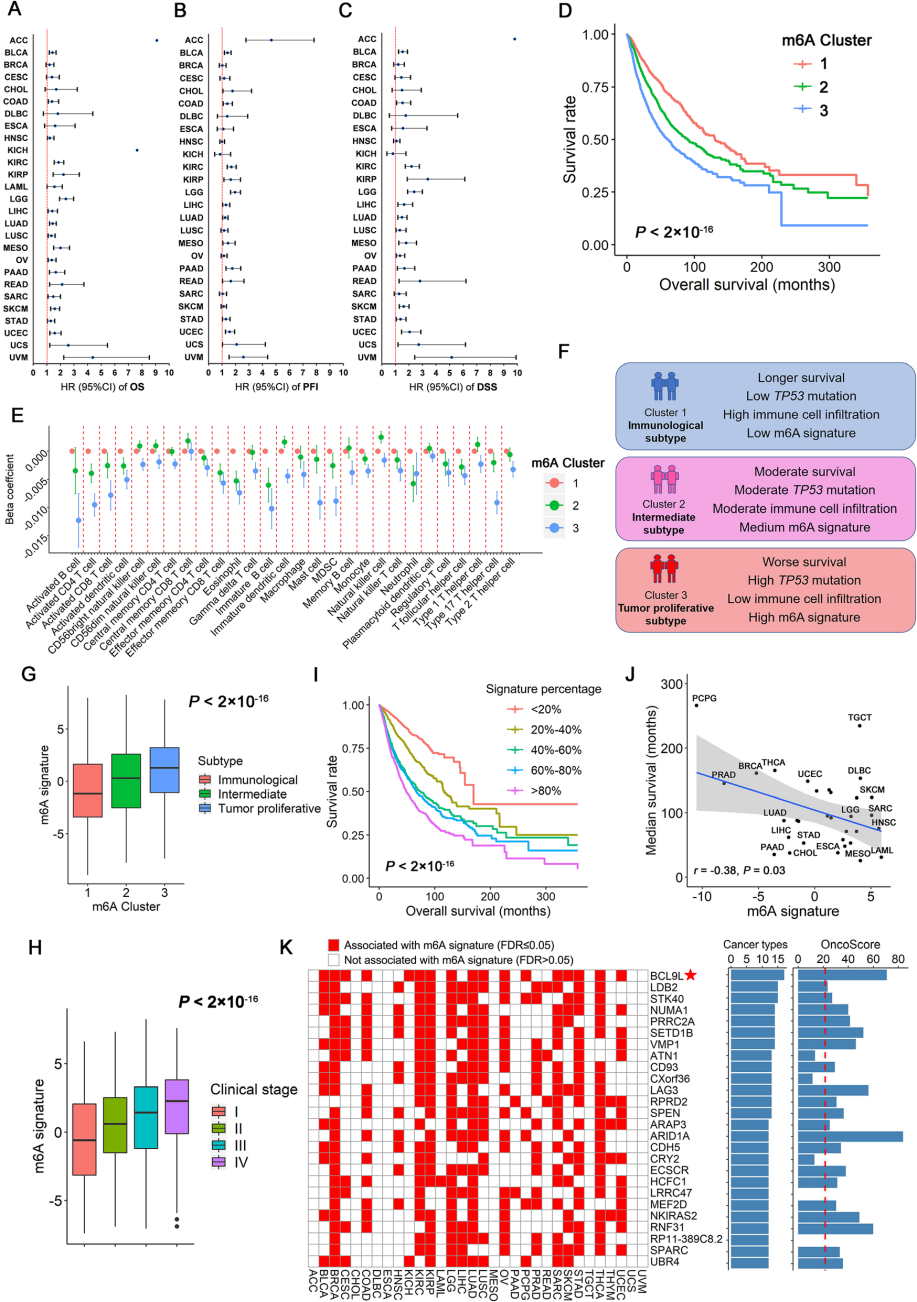
**a**-**c** Associations of the m6A subtypes and pan-cancer overall survival (OS), Progression-free interval (PFI) and disease-specific survival (DSS). The hazard ratios (HR) are evaluated by the trend association (clusters 1–3) in the Cox proportional hazard models. **d** Kaplan-Meier plots of the m6A subtypes and overall survival. **e** Associations between m6A subtypes and the ssGSEA scores of tumor microenvironment cell infiltration. Cluster 1 is used as a reference group. **f** Summary of the characteristics of different m6A subtypes. **g** Correlation of the m6A signature and m6A subtypes. **h** Distributions of the m6A signature across different clinical stage (I-IV). **i** Kaplan-Meier plot of the m6A signature and overall survival. We categorized the signature into five subgroups with equal sample sizes and adjusted the curves with age, gender, stage, cancer types and probable estimations of expression residual (PEER) factors. **j** Correlation plot of the m6A signature and median survival time (MST) of each cancer type. **k** The top potential m6A targets associated with the m6A signature in ≥12 cancer types. We used the OncoScore system to evaluate their relationships with cancer based on previous literature reports

We used the K-means algorithm to categorize patients into different m6A subtypes (clusters) within each cancer type, separately. Three subtypes were identified by the Elbow method (Figures S4-S5). The log-rank test detected that the defined subtypes were significantly associated with overall survival in 24 of 27 cancers after excluding cancers with death proportion < 10% (Figure S6). Specifically, we defined the clusters sorted by median survival time (MST). The group with the longest MST was defined as cluster 1, while the group with the shortest MST was defined as cluster 3, and the middle group was cluster 2. Compared with cluster 1, clusters 2 and 3 had significantly worse survival in 22 of 27 cancers (*P*_*trend*_ < 0.05 in Cox proportional-hazards model) (Fig. 2a). In addition, the classifiers remained significant in most cancer types when the clinical outcome was progression-free interval (PFI) (16 of 26) (Fig. 2b) or disease-specific survival (DSS) (18 of 26) (Fig. 2c). In the Kaplan-Meier survival analysis of overall population, m6A subtypes could stratify patients’ survival significantly after adjusting for cancer types (*P* < 2 × 10^− 16^) (Fig. 2d). Distributions of four somatic mutations were significantly different among different m6A subtypes (FDR-correct *P* values of Chi-square test < 0.05), including *TP53, NOTCH1, CTNNB1*, and *PTEN* (Figure S7).

In the analysis of single sample gene set enrichment analysis (ssGSEA) of tumor microenvironment (TME) cell infiltration, the beta coefficients (95% CIs) of clusters 2 and 3 were shown in Fig. 2e while cluster 1 was used as the reference group. A total of 27 of 28 immune categories showed significant differences between m6A subtypes after FDR correction except the central memory CD8 T cell category. Cluster 3 had the lowest TME infiltration degree while cluster 1 had the highest. Thus, we defined cluster 1 as an immunological subtype, cluster 2 as an intermediate subtype, and cluster 3 as tumor proliferative subtype (Fig. 2f).

The PCA-generated m6A signature (Additional file: Methods, Figure S8) was significantly different across different m6A subtypes (trend in linear regression: β = 0.8, 95% CI: 0.68–0.91, *P* = 8.66 × 10^− 44^) (Fig. 2g). Higher level of m6A signature was significantly associated with worse overall survival in the overall population after adjusting for age, gender, stage, cancer types and probable estimations of expression residual (PEER) factors (trend in Cox regression: *P* = 2.07 × 10^− 86^) (Additional file: Methods, Figure 2I). In addition, the m6A signature was significantly higher among patients with late clinical stage disease (*P*_*trend*_ = 6.37 × 10^− 83^) or high tumor grade (*P* = 8.16 × 10^− 23^) (Figure 2h, S9). Higher m6A signature was significantly associated with shorter MST across different cancer types (*r* = − 0.38, *P* = 0.030) (Fig. 2j).

We checked the associations between the m6A signature and tumor mutation burden (TMB) score in the overall pan-cancer population. Unsurprisingly, they had a strong positive correlation with Pearson *r* = 0.53 in the overall population (Figure S10A). The m6A signature was positively correlated (*P*_*FDR*_ < 0.05) with the TMB scores in 16 cancer types (Figure S10B).

We applied the ssGSEA to compute the enrichment score of 2236 canonical pathways across the genome. A total of 949 pathways (42.4%) were significantly associated with the m6A signature after FDR corrections, indicating that m6A modifications are linked to a broad range of biological processes (Table S2), with consistent results across most of cancer types (Figure S11). Also identified were some classical pathways related to cancer therapy, such as the FOXM1 pathway, cell cycle regulation, polo-like kinase 1 (PLK1)-related pathways, and Aurora A/B pathways.

### Potential targets that interact with m6A modification

We identified 114 novel genes associated with the signature in at least 10 cancer subtypes with *P*_*FDR*_ < 0.05 (Fig. 2k). Among the 105 genes with available OncoScores (Additional file: Methods), 78 genes (74.3%) passed the suggested threshold (OncoScore> 21.09), which was much higher than the proportion of randomly selected oncogenes (35%) in the OncoScore evaluation system (*χ*^2^ = 67.3, *P* = 2.29 × 10^− 16^) (Table S3).

The gene with the highest frequency of significance across cancers was a protein-coding gene, *BCL9L* (17/32 cancer types, OncoScore = 70.76). *BCL9L* was significantly up-regulated in tumor tissues in 10 cancer types (Figure S12). Its expression levels were positively associated with the m6A signature (*r* = 0.35) and m6A subtypes (*P*_*trend*_ = 2.81 × 10^− 66^) in the overall population (Figure S13A, S13B). Higher expression of *BCL9L* was significantly associated with worse overall survival in the meta-analysis of pan-cancer (HR = 1.14, 95% CI: 1.07–1.24, *P* = 0.0002) (Figure S13C). This finding was confirmed in six external validation datasets, including gene expression datasets of lung cancer (HR = 1.32, *P* = 9.00 × 10^− 4^), gastric cancer (HR = 1.62, *P* = 1.32 × 10^− 5^), breast cancer (HR = 1.38, *P* = 0.048), liver cancer (HR = 1.44, *P* = 0.037), and ovarian cancer (HR = 1.45, *P* = 1.20 × 10^− 4^) and a protein dataset of breast cancer (HR = 3.58, *P* = 7.00 × 10^− 4^) (Figure S13D and S14). *BCL9L* is an important member of the Wnt signaling pathway, and is significantly associated with the ssGSEA enrichment score of the Wnt pathway (*r* = 0.39) (Figure S13E). Among the five m6A interactive genes (*MYC, LEF1, WIF1, CTNNB1*, and *SOX2*) that also participate in the Wnt signaling progress, *BCL9L* had strong correlations with *MYC* (*r* = 0.34) and *CTNNB1* (*r* = 0.36) (Figure S15).

Further, we validated the 109 protein-coding genes in the external datasets. Forty genes (37.7%) were associated with survival in the meta-analysis with *P*_*FDR*_ < 0.05, indicating they played important roles in cancers (Table S4).

## Conclusions

In summary, this study demonstrates that m6A regulators and interactive genes may play an important role in cancer outcomes. Our systemic evaluation of m6A patterns improves the understanding of the dysregulation of RNA methylation in tumor microenvironments. The predicted interactive target genes may provide additional insight into clinical therapeutic targets.

## Supplementary Information

**Additional file 1. Supplementary materials and methods, and supplementary Table S1-S7, Figure S1-S15. Table S1. **Summary of genes that interact with m6A modification**. Table S2. **Top 200 canonical pathways associated with the m6A signature.** Table S3. **Potential m6A modification targets associated with the m6A signature.** Table S4. **Independent validation of the potential m6A targets. **Table S5. **Study characteristics of the pan-cancer datasets.** Table S6. **Sample size of tumor-normal pairs in each cancer type.** Table S7. **Gene list used to generate the m6A subtypes in each cancer.** Figure S1. **Heatmap of the fold change (FC) values in pan-cancer.** Figure S2. **PCA plots of m6A related genes in tumors and adjacent normal tissues.** Figure S3. **Receiver-operating characteristic curves of predicted performance to discriminate from tumor and normal tissues. **Figure S4. **Average cost of different clusters using the Elbow method.** Figure S5. **Distribution of the m6A subtype across each cancer type.** Figure S6. **Kaplan-Meier plots of m6A subtypes and overall survival in pan-cancer. **Figure S7. **Associations between somatic mutations and m6A subtypes. **Figure S8. **Heatmap of the genes associated with overall survival in at least five cancer types and used to generate an m6A signature. **Figure S9. **Distribution of m6A signature in low-grade (grade 1&2) and high-grade (grade 3&4) patients.** Figure S10. **Associations between tumor mutation burden score and m6A signature in pan-cancer.** Figure S11. **The top 20 biological pathways that are associated with the m6A signature.** Figure S12. **Differential comparison of BCL9L in TCGA tumor and adjacent-normal tissues.** Figure S13. **BCL9L and survival outcome in pan-cancer.** Figure S14. **External validation of BCL9L prognostic value in public GEO datasets.** Figure S15. **Correlations between BCL9L and m6A interactive genes in the Wnt signaling pathway. (DOCX 7598kb)

## Data Availability

TCGA Data Poral: https://portal.gdc.cancer.gov/ GEO Datasets: https://www.ncbi.nlm.nih.gov/gds/ KM-plotter: http://kmplot.com/analysis/ MSigDB: https://www.gsea-msigdb.org/gsea/msigdb/index.jsp

## Abbreviations

m6A: N6-Methyladenosine
TCGA: The Cancer Genome Atlas
FPKM: Fragments per kilobase per million
RPM: Reads per million miRNA mapped
TME: Tumor microenvironment
ssGSEA: Single sample gene set enrichment analysis
GEO: Gene Expression Omnibus
ROC: Receiver-operating characteristic
AUC: Area under the curve
MST: Median survival time
DSS: Disease-specific survival
PFI: Progression-free interval
TMB: Tumor mutation burden
PCA: Principal component analysis
