# Supplementary material for: Comprehensive analyses of m6A regulators and interactive coding and non-coding RNAs across 32 cancer types
Source: Mol Cancer. 2021 Apr 13;20:67. doi: 10.1186/s12943-021-01362-2 (PMC8045265; doi:10.1186/s12943-021-01362-2)
Supplement: Supplementary file 1 — Additional file 1. Supplementary materials and methods, and supplementary Table S1-S7, Figure S1-S15. Table S1. Summary of genes that interact with m6A modification. Table S2. Top 200 canonical pathways associated with the m6A signature. Table S3. Potential m6A modification targets associated with the m6A signature. Table S4. Independent validation of the potential m6A targets. Table S5. Study characteristics of the pan-cancer datasets. Table S6. Sample size of tumor-normal pairs in each cancer type. Table S7. Gene list used to generate the m6A subtypes in each cancer. Figure S1. Heatmap of the fold change (FC) values in pan-cancer. Figure S2. PCA plots of m6A related genes in tumors and adjacent normal tissues. Figure S3. Receiver-operating characteristic curves of predicted performance to discriminate from tumor and normal tissues. Figure S4. Average cost of different clusters using the Elbow method. Figure S5. Distribution of the m6A subtype across each cancer type. Figure S6. Kaplan-Meier plots of m6A subtypes and overall survival in pan-cancer. Figure S7. Associations between somatic mutations and m6A subtypes. Figure S8. Heatmap of the genes associated with overall survival in at least five cancer types and used to generate an m6A signature. Figure S9. Distribution of m6A signature in low-grade (grade 1&2) and high-grade (grade 3&4) patients. Figure S10. Associations between tumor mutation burden score and m6A signature in pan-cancer. Figure S11. The top 20 biological pathways that are associated with the m6A signature. Figure S12. Differential comparison of BCL9L in TCGA tumor and adjacent-normal tissues. Figure S13. BCL9L and survival outcome in pan-cancer. Figure S14. External validation of BCL9L prognostic value in public GEO datasets. Figure S15. Correlations between BCL9L and m6A interactive genes in the Wnt signaling pathway. (DOCX 7598kb) [file 12943_2021_1362_MOESM1_ESM.docx]

# Comprehensive analyses of m6A regulators and interactive coding and non-coding RNAs across 32 cancer types

# Supplementary Information

[Methods 3](#_Toc66718366)

[Reference 6](#_Toc66718367)

[Table S1. Summary of genes that interact with m6A modification 7](#_Toc66718368)

[Table S2. Top 200 canonical pathways associated with the m6A signature 9](#_Toc66718369)

[Table S3. Potential m6A modification targets associated with the m6A signature 15](#_Toc66718370)

[Table S4. Independent validation of the potential m6A targets 18](#_Toc66718371)

[Table S5. Study characteristics of the pan-cancer datasets 24](#_Toc66718372)

[Table S6. Sample size of tumor-normal pairs in each cancer type. 25](#_Toc66718373)

[Table S7. Gene list used to generate the m6A subtypes in each cancer 26](#_Toc66718374)

[Figure S1. Heatmap of the fold change (FC) values in pan-cancer. 28](#_Toc66718375)

[Figure S2. PCA plots of m6A related genes in tumors and adjacent normal tissues 29](#_Toc66718376)

[Figure S3. Receiver-operating characteristic curves of predicted performance to discriminate from tumor and normal tissues 30](#_Toc66718377)

[Figure S4. Average cost of different clusters using the Elbow method. 31](#_Toc66718378)

[Figure S5. Distribution of the m6A subtype across each cancer type 32](#_Toc66718379)

[Figure S6. Kaplan-Meier plots of m6A subtypes and overall survival in pan-cancer 33](#_Toc66718380)

[Figure S7. Associations between somatic mutations and m6A subtypes. 34](#_Toc66718381)

[Figure S8. Heatmap of the genes associated with overall survival in at least five cancer types and used to generate an m6A signature 35](#_Toc66718382)

[Figure S9. Distribution of m6A signature in low-grade (grade 1&2) and high-grade (grade 3&4) patients 36](#_Toc66718383)

[Figure S10. Associations between tumor mutation burden score and m6A signature in pan-cancer 37](#_Toc66718384)

[Figure S11. The top 20 biological pathways that are associated with the m6A signature. 38](#_Toc66718385)

[Figure S12. Differential comparison of](#_Toc66718386) *[BCL9L](#_Toc66718386)* [in TCGA tumor and adjacent-normal tissues 39](#_Toc66718386)

[Figure S13. BCL9L and survival outcome in pan-cancer 40](#_Toc66718387)

[Figure S14. External validation of](#_Toc66718388) *[BCL9L](#_Toc66718388)* [prognostic value in public GEO datasets 41](#_Toc66718388)

[Figure S15. Correlations between](#_Toc66718389) *[BCL9L](#_Toc66718389)* [and m6A interactive genes in the Wnt signaling pathway 42](#_Toc66718389)

## Methods

***Collection of m6A regulators and interactive genes***

A total of 23 m6A regulators were selected in this study (1), including eight writers (*METTL3, METTL14, METTL16, RBM15, RBM15B, WTAP, KIAA1429, ZC3H13*), three erasers (*ALKBH5, ALKBH3, FTO*) and 12 readers (*YTHDC1, YTHDC2, YTHDF1, YTHDF2, YTHDF3, IGF2BP1, IGF2BP2, IGF2BP3, HNRNPA2B1, HNRNPC, RBMX, EIF3A*). We also collected 56 protein-coding genes, 10 lncRNAs, and 17 miRNAs that interact with m6A modifications, mainly based on the literature evidence (Table S1). All genes were mapped to Ensembl IDs.

***Pan-cancer multi-omics data source and preprocessing***

Our analyses were based on gene expression profiles and miRNA expression profiles generated by the The Cancer Genome Atlas (TCGA) Research Network, and 33 unique TCGA cancer projects were downloaded. Gene expression data including protein-coding mRNAs and lncRNAs were downloaded from the RNA sequencing platform and normalized to fragments per kilobase per million (FPKM) values. miRNA data were downloaded from the miRNA sequencing platform and normalized to reads per million miRNA mapped (RPM) values. All gene features (e.g., chromosome positions, gene types, Ensembl IDs, official symbols) were defined by the GENCODE project (version 25) on hg 19 coordinate (2). Clinical information including age, gender, clinical stage, overall survival, and death status was downloaded from the TCGA project (3).

A total of 10,173 samples with gene expression and 10,121 samples with miRNA expression were downloaded. After removing observations without complete information, 9,804 cases with complete gene expression, miRNA expression, and clinical information remained with 32 cancer types included (Table S5).

In addition, 698 pairs of tumor and adjacent-normal tissues had gene expression data, while 655 pairs had miRNAs expression data. Comparisons of expression data between tumor and normal tissues were conducted only in cancers with at least three tumor-normal pairs. Included in analyses were a total of 592 pairs with both gene expression and miRNA data (Table S6).

All expression values were log-transformed. The ComBat method was used to adjust for batch effects between different cancer types using the R package *sva* (4). We also calculated the 15 most probable estimations of expression residual (PEER) factors as covariates to account for potential confounders (5).

Somatic mutation data from whole exome/genome sequencing (WXS/WGS) were download from the GDC Pan-Cancer project on the USCS Xena Sever (6). Data processing was followed by utilization of the GDC DNA-Seq analysis pipeline. SNVs were aggregated and masked by the MuTect2 algorithm (7).

***Construction of gene co-expression network***

We used weighted correlation network analysis (WGCNA) to investigate the co-expression relationship among m6A interactive genes (8). Gene pairs with Pearson correlation coefficients ≥0.3 were selected to build the co-expression network, which was drawn using Cytoscape 3.8.0 software.

***Discrimination of tumor and adjacent normal tissues***

In comparative analysis of tumor and adjacent normal tissues, the fold change (FC) was calculated as $FC\text{=}\frac{\text{Mean expression}_{\text{tumor tissue}}}{\text{Mean expression}_{\text{nomal tissue}}}$. We used principal component analysis (PCA) to compute the top two PCs of the selected subset population and generate the score as $score\text{ }=\text{ }PC_{1}+PC_{2}$. The score was further evaluated by the receiver operating characteristic curve (ROC) to discriminate tumor from normal tissues.

***Tumor mutation burden estimation***

The tumor mutation burden (TMB) is a crucial biomarker in cancer immunotherapy (9) and was computed as (10):

$$\begin{matrix} TMB\text{ }score\text{ }=\text{ }total\text{ }number\text{ }of\text{ }truncating\text{ }mutations\times2.0 + \\ \text{ }total\text{ }number\text{ }of\text{ }non-truncating\text{ }mutations\times1.0 \end{matrix}$$

where nonsense, frameshift indels, and splice-site mutations were included in the truncating mutation category while missense, in-frame indels, and nonstop mutations were included in the non-truncating mutation category.

***Unsupervised approach to define m6A subtypes***

Based on the distinct m6A patterns, we classified patients into different subgroups within each cancer subtype using K-means clustering analysis. Specifically, we used the Hartigan–Wong algorithm with 1,000 iterations to ensure the stability of classification. The number of clusters was determined by the Elbow method (11). The subtypes were generated using a group of differential expressed genes within each cancer type, separately. The genes that differential expressed between tumor and normal tissues (FC>1.5 or FC<0.67 and *P*_FDR_<0.05 by paired student t-test) were included in the K-means algorithm. For those cancer types with no data available from normal tissues, all the genes above were used to calculate the m6A subtypes (Table S7).

***Unsupervised approach to define m6A signature***

To quantify m6A modification patterns at the individual level, we developed an m6A signature based on a two-step approach. First, we selected genes by examining their individual associations with survival using the Cox regression model after adjusting for age, gender, and clinical stage. Risk genes and protective genes with nominal *P*<0.05 in at least five cancer types were selected as potential signals. Second, we used PCA to construct the m6A signature (12, 13). We calculated the signature as: , where $PC_{1i}$ and $PC_{2i}$ referred to the first *PC* of the risk and protective gene sets, respectively.

To evaluate the association between the signature and survival, we categorized the signature into categorical variable with five classifications (Coded as 1: <20%, 2: 20%-40%, 3: 40%-60%, 4: 60%-80%, 5: >80%). The *P* value of trend was generated by the Cox regression model adjusting for age, gender, stage, cancer types and probable estimations of expression residual (PEER) factors.

***Tumor microenvironment cell infiltration estimation***

m6A modification is associated with tumor microenvironment (TME) infiltration (13). We collected 28 human TME infiltration categories, such as activated B cells, activated CD4 T cells, macrophages, natural killer cells, and neutrophils. The gene sets of each TME infiltration immune cell types were obtained from Charoentong et. al. (14).

To determine the degree of enrichment in different categories, we used single sample gene set enrichment analysis (ssGSEA) to calculate the enrichment score of each immune category for each individual (15). The scores were utilized to represent the abundance of TME immune infiltrating cells in each individual. ssGSEA analysis was performed using the R package *GSVA*.

***Canonical biological pathways enrichment***

To identify biological pathways related to m6A modification patterns, we collected 2,232 canonical pathways compiled by domain experts including BIOCARTA, KEGG, PID, and REACTOME reference databases, which were downloaded from the MSigDB database (c2.cp.v7.1.symbols.gmt) (16). ssGSEA was used to calculate the enrichment score of each pathway.

***Literature evidence for the identified target genes in cancer***

We used OncoScore, a text mining tool to assess the associations between each gene and specific cancers based on the literature. A cutoff value of 21.09 was suggested to determine true positives and the true negatives in cancer gene identification (17).

***External independent validation of BCL9L and cancer prognosis***

To validate the association between the selected target genes and survival, we integrated multiple public datasets of primary tumor tissues in Gene Expression Omnibus (GEO) for lung cancer (n=1144), gastric cancer (n=631), breast cancer (n=626 for mRNA, n=65 for protein), liver cancer (n=364), and ovarian cancer (n=614) from the Kaplan-Meier Plotter sever (18). Gene expression levels were dichotomized into high expression and low expression groups using the median value within each dataset.

***Methods for survival analysis and survival prediction***

Kaplan-Meier survival curves were drawn and compared among subgroups using Cox proportional hazards mode and stratified by cancer types. We used the Cox proportional hazard model to visualize the association between survival and multiple predictors.

All statistical analyses were performed using R version 3.6.3 (The R Foundation). *P* values were two-sided, and *P* < 0.05 (or FDR < 0.05 with multiple comparisons) was considered statistically significant.

## Reference

1. Chen XY, Zhang J, Zhu JS. The role of m(6)A RNA methylation in human cancer. Mol Cancer. 2019;18(1):103.

2. Frankish A, Diekhans M, Ferreira AM, Johnson R, Jungreis I, Loveland J, et al. GENCODE reference annotation for the human and mouse genomes. Nucleic Acids Res. 2019;47(D1):D766-d73.

3. Liu J, Lichtenberg T, Hoadley KA, Poisson LM, Lazar AJ, Cherniack AD, et al. An Integrated TCGA Pan-Cancer Clinical Data Resource to Drive High-Quality Survival Outcome Analytics. Cell. 2018;173(2):400-16 e11.

4. Johnson WE, Li C, Rabinovic A. Adjusting batch effects in microarray expression data using empirical Bayes methods. Biostatistics. 2007;8(1):118-27.

5. Stegle O, Parts L, Piipari M, Winn J, Durbin R. Using probabilistic estimation of expression residuals (PEER) to obtain increased power and interpretability of gene expression analyses. Nat Protoc. 2012;7(3):500-7.

6. Goldman MJ, Craft B, Hastie M, Repecka K, McDade F, Kamath A, et al. Visualizing and interpreting cancer genomics data via the Xena platform. Nat Biotechnol. 2020;38(6):675-8.

7. Cibulskis K, Lawrence MS, Carter SL, Sivachenko A, Jaffe D, Sougnez C, et al. Sensitive detection of somatic point mutations in impure and heterogeneous cancer samples. Nat Biotechnol. 2013;31(3):213-9.

8. Langfelder P, Horvath S. WGCNA: an R package for weighted correlation network analysis. BMC Bioinformatics. 2008;9:559.

9. Chan TA, Yarchoan M, Jaffee E, Swanton C, Quezada SA, Stenzinger A, et al. Development of tumor mutation burden as an immunotherapy biomarker: utility for the oncology clinic. Ann Oncol. 2019;30(1):44-56.

10. Wang X, Li M. Correlate tumor mutation burden with immune signatures in human cancers. BMC Immunol. 2019;20(1):4.

11. Kodinariya TM, Makwana PR. Review on determining number of Cluster in K-Means Clustering. International Journal. 2013;1(6):90-5.

12. Zeng D, Li M, Zhou R, Zhang J, Sun H, Shi M, et al. Tumor Microenvironment Characterization in Gastric Cancer Identifies Prognostic and Immunotherapeutically Relevant Gene Signatures. Cancer Immunol Res. 2019;7(5):737-50.

13. Zhang B, Wu Q, Li B, Wang D, Wang L, Zhou YL. m(6)A regulator-mediated methylation modification patterns and tumor microenvironment infiltration characterization in gastric cancer. Mol Cancer. 2020;19(1):53.

14. Charoentong P, Finotello F, Angelova M, Mayer C, Efremova M, Rieder D, et al. Pan-cancer Immunogenomic Analyses Reveal Genotype-Immunophenotype Relationships and Predictors of Response to Checkpoint Blockade. Cell Rep. 2017;18(1):248-62.

15. Shen S, Wang G, Zhang R, Zhao Y, Yu H, Wei Y, et al. Development and validation of an immune gene-set based Prognostic signature in ovarian cancer. EBioMedicine. 2019;40:318-26.

16. Liberzon A, Birger C, Thorvaldsdottir H, Ghandi M, Mesirov JP, Tamayo P. The Molecular Signatures Database (MSigDB) hallmark gene set collection. Cell Syst. 2015;1(6):417-25.

17. Piazza R, Ramazzotti D, Spinelli R, Pirola A, De Sano L, Ferrari P, et al. OncoScore: a novel, Internet-based tool to assess the oncogenic potential of genes. Scientific reports. 2017;7:46290.

18. Gyorffy B, Lanczky A, Szallasi Z. Implementing an online tool for genome-wide validation of survival-associated biomarkers in ovarian-cancer using microarray data from 1287 patients. Endocr Relat Cancer. 2012;19(2):197-208.

### Table S1. Summary of genes that interact with m6A modification

| Chr | Gene type | Gene symbol | Gene ID | QC* | PMID |
| --- | --- | --- | --- | --- | --- |
| chr1 | protein_coding | *ZMYM1* | ENSG00000197056 | PASS | 31607270 |
| chr1 | protein_coding | *SRSF11* | ENSG00000116754 | PASS | 31530567 |
| chr1 | protein_coding | *LAMTOR5* | ENSG00000134248 | PASS | 30181579 |
| chr1 | protein_coding | *HDGF* | ENSG00000143321 | PASS | 31582403 |
| chr1 | antisense | *GAS5-AS1* | ENSG00000270084 | PASS | 31497208 |
| chr1 | processed_transcript | *GAS5* | ENSG00000234741 | PASS | 31206689 |
| chr2 | protein_coding | *ID2* | ENSG00000115738 | PASS | 31118692 |
| chr2 | protein_coding | *CXCR4* | ENSG00000121966 | PASS | 31239444 |
| chr2 | protein_coding | *ITGA6* | ENSG00000091409 | PASS | 31409574 |
| chr2 | protein_coding | *PDCD1* | ENSG00000188389 | PASS | 31239444 |
| chr2 | miRNA | *miR-4429* | ENSG00000264010 | FAIL | 31395342 |
| chr2 | miRNA | *miR-375* | ENSG00000198973 | PASS | 31839484 |
| chr2 | miRNA | *miR-1246* | ENSG00000283203 | FAIL | 31492150 |
| chr3 | protein_coding | *CTNNB1* | ENSG00000168036 | PASS | 31870368 |
| chr3 | protein_coding | *CDCP1* | ENSG00000163814 | PASS | 30796352 |
| chr3 | protein_coding | *SEC62* | ENSG00000008952 | PASS | 31395342 |
| chr3 | protein_coding | *SOX2* | ENSG00000181449 | PASS | 31230592 |
| chr3 | miRNA | *let-7g* | ENSG00000199150 | PASS | 29174803 |
| chr4 | processed_transcript | *DANCR* | ENSG00000226950 | PASS | 31804607 |
| chr4 | protein_coding | *LEF1* | ENSG00000138795 | PASS | 31253399 |
| chr5 | protein_coding | *AFF4* | ENSG00000072364 | PASS | 30659266 |
| chr5 | protein_coding | *ADAM19* | ENSG00000135074 | PASS | 28297667 |
| chr5 | miRNA | *miR-143* | ENSG00000284182 | PASS | 30464631 |
| chr5 | miRNA | *miR-145* | ENSG00000276365 | PASS | 28104805 |
| chr6 | protein_coding | *SRSF3* | ENSG00000112081 | PASS | 26876937 |
| chr6 | protein_coding | *SRF* | ENSG00000112658 | PASS | 30371874 |
| chr6 | protein_coding | *MYB* | ENSG00000118513 | PASS | 29290617 |
| chr7 | protein_coding | *FSCN1* | ENSG00000075618 | PASS | 29476152 |
| chr7 | protein_coding | *EGFR* | ENSG00000146648 | PASS | 30423408 |
| chr7 | miRNA | *miR-29a* | ENSG00000226380 | PASS | 31169310 |
| chr7 | miRNA | *miR-25* | ENSG00000207547 | PASS | 31015415 |
| chr7 | miRNA | *miR-106b* | ENSG00000208036 | PASS | 28600480 |
| chr7 | miRNA | *miR-29b-1* | ENSG00000283797 | PASS | 31263129 |
| chr7 | miRNA | *miR-671* | ENSG00000284191 | PASS | 29887379 |
| chr8 | protein_coding | *IKBKB* | ENSG00000104365 | PASS | 30659266 |
| chr8 | protein_coding | *MYC* | ENSG00000136997 | PASS | 28920958 |
| chr9 | protein_coding | *NOTCH1* | ENSG00000148400 | PASS | 31760940 |
| chr9 | miRNA | *miR-126* | ENSG00000199161 | PASS | 29371906 |
| chr10 | lincRNA | *GATA3-AS1* | ENSG00000197308 | PASS | 30420860 |
| chr10 | protein_coding | *GATA3* | ENSG00000107485 | PASS | 31856849 |
| chr10 | protein_coding | *PTEN* | ENSG00000171862 | PASS | 28920958 |
| chr10 | protein_coding | *BNIP3* | ENSG00000176171 | PASS | 30922314 |
| chr11 | lincRNA | *LINC00958* | ENSG00000251381 | PASS | 31915027 |
| chr11 | lincRNA | *NEAT1* | ENSG00000245532 | PASS | 31290116 |
| chr11 | lincRNA | *MALAT1* | ENSG00000251562 | PASS | 27872311 |
| chr11 | protein_coding | *RELA* | ENSG00000173039 | PASS | 30659266 |
| chr11 | protein_coding | *YAP1* | ENSG00000137693 | PASS | 29180467 |
| chr11 | protein_coding | *ETS1* | ENSG00000134954 | PASS | 31438961 |
| chr11 | miRNA | *miR-125b-1* | ENSG00000207971 | PASS | 25451164 |
| chr12 | protein_coding | *FOXM1* | ENSG00000111206 | PASS | 28344040 |
| chr12 | protein_coding | *NANOG* | ENSG00000111704 | PASS | 27001847 |
| chr12 | protein_coding | *SP1* | ENSG00000185591 | PASS | 29186125 |
| chr12 | protein_coding | *GLI1* | ENSG00000111087 | PASS | 31806999 |
| chr12 | protein_coding | *WIF1* | ENSG00000156076 | PASS | 31906946 |
| chr12 | protein_coding | *SOCS2* | ENSG00000120833 | PASS | 29171881 |
| chr14 | antisense | *ARHGAP5-AS1* | ENSG00000258655 | PASS | 31097692 |
| chr14 | protein_coding | *HIF1A* | ENSG00000100644 | PASS | 26996300 |
| chr14 | protein_coding | *ASB2* | ENSG00000100628 | PASS | 28017614 |
| chr15 | miRNA | *miR-1268a* | ENSG00000221641 | FAIL | 31263129 |
| chr15 | miRNA | *miR-1266* | ENSG00000221052 | PASS | 31263129 |
| chr16 | protein_coding | *USP7* | ENSG00000187555 | PASS | 30905413 |
| chr16 | protein_coding | *PHLPP2* | ENSG00000040199 | PASS | 30154548 |
| chr17 | protein_coding | *TP53* | ENSG00000141510 | PASS | 30578766 |
| chr17 | protein_coding | *RARA* | ENSG00000131759 | PASS | 28017614 |
| chr17 | protein_coding | *SP2* | ENSG00000167182 | PASS | 29186125 |
| chr17 | protein_coding | *TK1* | ENSG00000167900 | PASS | 29476152 |
| chr17 | miRNA | *miR-21* | ENSG00000284190 | PASS | 26957558 |
| chr17 | miRNA | *miR-744* | ENSG00000266297 | PASS | 29120412 |
| chr18 | protein_coding | *BCL2* | ENSG00000171791 | PASS | 28920958 |
| chr19 | protein_coding | *KEAP1* | ENSG00000079999 | PASS | 31653849 |
| chr19 | protein_coding | *BRD4* | ENSG00000141867 | PASS | 30232453 |
| chr19 | protein_coding | *CEBPA* | ENSG00000245848 | PASS | 29249359 |
| chr19 | protein_coding | *AXL* | ENSG00000167601 | PASS | 30249526 |
| chr19 | protein_coding | *IL11* | ENSG00000095752 | PASS | 31735169 |
| chr19 | protein_coding | *MZF1* | ENSG00000099326 | PASS | 29842885 |
| chr20 | protein_coding | *E2F1* | ENSG00000101412 | PASS | 31827395 |
| chr20 | protein_coding | *SRSF6* | ENSG00000124193 | PASS | 31530567 |
| chr20 | antisense | *KCNK15-AS1* | ENSG00000244558 | PASS | 30032148 |
| chr20 | protein_coding | *UBE2C* | ENSG00000175063 | PASS | 29904125 |
| chr20 | protein_coding | *SNAI1* | ENSG00000124216 | PASS | 30772441 |
| chr22 | protein_coding | *P2RX6* | ENSG00000099957 | PASS | 31159832 |
| chr22 | miRNA | *miR-33a* | ENSG00000207932 | PASS | 27856248 |
| chr22 | protein_coding | *SOX10* | ENSG00000100146 | FAIL | 31239444 |
| chrX | lincRNA | *XIST* | ENSG00000229807 | PASS | 27602518 |
| chrX | protein_coding | *TAZ* | ENSG00000102125 | PASS | 27117702 |
| chrX | miRNA | *miR-221* | ENSG00000207870 | PASS | 31228940 |
| chrX | miRNA | *miR-222* | ENSG00000207725 | PASS | 31228940 |

*In the quality control (QC) procedure, low expressed genes (more than half were zero or mean value < 0.1) were excluded.

### Table S2. Top 200 canonical pathways associated with the m6A signature

|  | Pathway | Beta | Se | Z score |
| --- | --- | --- | --- | --- |
| 1 | PID_FOXM1_PATHWAY | 0.010 | 8.40E-05 | 113.6 |
| 2 | KEGG_CELL_CYCLE | 0.006 | 5.73E-05 | 107.3 |
| 3 | REACTOME_POLO_LIKE_KINASE_MEDIATED_EVENTS | 0.014 | 1.32E-04 | 106.8 |
| 4 | BIOCARTA_RANMS_PATHWAY | 0.009 | 8.30E-05 | 106.7 |
| 5 | REACTOME_CONDENSATION_OF_PROMETAPHASE_CHROMOSOMES | 0.013 | 1.28E-04 | 104.9 |
| 6 | REACTOME_RHO_GTPASES_ACTIVATE_FORMINS | 0.006 | 6.11E-05 | 104.7 |
| 7 | PID_AURORA_B_PATHWAY | 0.010 | 9.83E-05 | 104.2 |
| 8 | PID_PLK1_PATHWAY | 0.010 | 9.71E-05 | 103.6 |
| 9 | REACTOME_PHOSPHORYLATION_OF_EMI1 | 0.016 | 1.54E-04 | 103.0 |
| 10 | REACTOME_RESOLUTION_OF_SISTER_CHROMATID_COHESION | 0.008 | 7.46E-05 | 101.5 |
| 11 | REACTOME_G1_S_SPECIFIC_TRANSCRIPTION | 0.010 | 1.02E-04 | 100.4 |
| 12 | REACTOME_CELL_CYCLE_MITOTIC | 0.005 | 5.24E-05 | 99.3 |
| 13 | REACTOME_CYCLIN_A_B1_B2_ASSOCIATED_EVENTS_DURING_G2_M_TRANSITION | 0.009 | 9.41E-05 | 99.3 |
| 14 | REACTOME_G0_AND_EARLY_G1 | 0.009 | 8.98E-05 | 99.0 |
| 15 | REACTOME_MITOTIC_SPINDLE_CHECKPOINT | 0.007 | 7.46E-05 | 99.0 |
| 16 | PID_AURORA_A_PATHWAY | 0.006 | 6.27E-05 | 98.6 |
| 17 | REACTOME_CELL_CYCLE_CHECKPOINTS | 0.006 | 6.41E-05 | 98.2 |
| 18 | REACTOME_UNWINDING_OF_DNA | 0.013 | 1.33E-04 | 98.0 |
| 19 | BIOCARTA_MCM_PATHWAY | 0.010 | 9.82E-05 | 97.8 |
| 20 | REACTOME_CELL_CYCLE | 0.005 | 5.32E-05 | 96.8 |
| 21 | REACTOME_G2_M_DNA_REPLICATION_CHECKPOINT | 0.017 | 1.79E-04 | 96.4 |
| 22 | REACTOME_MITOTIC_METAPHASE_AND_ANAPHASE | 0.005 | 5.32E-05 | 95.4 |
| 23 | REACTOME_ACTIVATION_OF_NIMA_KINASES_NEK9_NEK6_NEK7 | 0.011 | 1.19E-04 | 95.4 |
| 24 | REACTOME_MITOTIC_G1_PHASE_AND_G1_S_TRANSITION | 0.005 | 5.33E-05 | 95.3 |
| 25 | REACTOME_MITOTIC_PROMETAPHASE | 0.006 | 6.24E-05 | 95.3 |
| 26 | REACTOME_TP53_REGULATES_TRANSCRIPTION_OF_CELL_CYCLE_GENES | 0.005 | 5.72E-05 | 95.2 |
| 27 | REACTOME_INITIATION_OF_NUCLEAR_ENVELOPE_NE_REFORMATION | 0.006 | 6.08E-05 | 94.6 |
| 28 | REACTOME_ACTIVATION_OF_THE_PRE_REPLICATIVE_COMPLEX | 0.010 | 1.06E-04 | 94.5 |
| 29 | REACTOME_SEPARATION_OF_SISTER_CHROMATIDS | 0.005 | 5.64E-05 | 93.9 |
| 30 | REACTOME_ACTIVATION_OF_ATR_IN_RESPONSE_TO_REPLICATION_STRESS | 0.010 | 1.05E-04 | 93.8 |
| 31 | REACTOME_DEPOSITION_OF_NEW_CENPA_CONTAINING_NUCLEOSOMES_AT_THE_CENTROMERE | 0.010 | 1.08E-04 | 93.4 |
| 32 | REACTOME_SUMOYLATION_OF_DNA_REPLICATION_PROTEINS | 0.006 | 6.11E-05 | 93.1 |
| 33 | PID_ATR_PATHWAY | 0.008 | 8.27E-05 | 92.7 |
| 34 | REACTOME_CDC6_ASSOCIATION_WITH_THE_ORC_ORIGIN_COMPLEX | 0.012 | 1.29E-04 | 90.8 |
| 35 | REACTOME_TP53_REGULATES_TRANSCRIPTION_OF_GENES_INVOLVED_IN_G2_CELL_CYCLE_ARREST | 0.006 | 6.70E-05 | 90.5 |
| 36 | REACTOME_M_PHASE | 0.004 | 4.88E-05 | 90.1 |
| 37 | REACTOME_DNA_STRAND_ELONGATION | 0.009 | 9.83E-05 | 89.9 |
| 38 | REACTOME_CHROMOSOME_MAINTENANCE | 0.007 | 8.11E-05 | 89.4 |
| 39 | REACTOME_CHK1_CHK2_CDS1_MEDIATED_INACTIVATION_OF_CYCLIN_B_CDK1_COMPLEX | 0.007 | 8.39E-05 | 89.3 |
| 40 | REACTOME_G2_M_CHECKPOINTS | 0.005 | 6.07E-05 | 89.1 |
| 41 | BIOCARTA_G2_PATHWAY | 0.007 | 7.61E-05 | 89.1 |
| 42 | REACTOME_TRANSCRIPTION_OF_E2F_TARGETS_UNDER_NEGATIVE_CONTROL_BY_P107_RBL1_AND_P130_RBL2_IN_COMPLEX_WITH_HDAC1 | 0.008 | 8.62E-05 | 88.1 |
| 43 | REACTOME_DNA_REPLICATION | 0.005 | 6.09E-05 | 87.8 |
| 44 | REACTOME_TP53_REGULATES_TRANSCRIPTION_OF_GENES_INVOLVED_IN_G1_CELL_CYCLE_ARREST | 0.010 | 1.16E-04 | 87.4 |
| 45 | REACTOME_RHO_GTPASE_EFFECTORS | 0.004 | 4.39E-05 | 86.2 |
| 46 | REACTOME_CONDENSATION_OF_PROPHASE_CHROMOSOMES | 0.009 | 1.02E-04 | 85.9 |
| 47 | REACTOME_E2F_ENABLED_INHIBITION_OF_PRE_REPLICATION_COMPLEX_FORMATION | 0.011 | 1.23E-04 | 85.5 |
| 48 | REACTOME_S_PHASE | 0.004 | 5.19E-05 | 85.3 |
| 49 | REACTOME_MITOTIC_G2_G2_M_PHASES | 0.004 | 4.30E-05 | 84.6 |
| 50 | REACTOME_E2F_MEDIATED_REGULATION_OF_DNA_REPLICATION | 0.007 | 8.33E-05 | 83.1 |
| 51 | REACTOME_GOLGI_CISTERNAE_PERICENTRIOLAR_STACK_REORGANIZATION | 0.005 | 6.55E-05 | 82.8 |
| 52 | REACTOME_TRANSCRIPTION_OF_E2F_TARGETS_UNDER_NEGATIVE_CONTROL_BY_DREAM_COMPLEX | 0.006 | 7.68E-05 | 82.7 |
| 53 | PID_MYC_ACTIV_PATHWAY | 0.004 | 4.71E-05 | 82.5 |
| 54 | REACTOME_DNA_REPLICATION_PRE_INITIATION | 0.005 | 6.05E-05 | 82.2 |
| 55 | REACTOME_HOMOLOGY_DIRECTED_REPAIR | 0.005 | 6.67E-05 | 82.1 |
| 56 | KEGG_HOMOLOGOUS_RECOMBINATION | 0.007 | 8.76E-05 | 81.4 |
| 57 | KEGG_DNA_REPLICATION | 0.007 | 8.07E-05 | 81.0 |
| 58 | REACTOME_DNA_DOUBLE_STRAND_BREAK_REPAIR | 0.005 | 5.87E-05 | 80.5 |
| 59 | PID_E2F_PATHWAY | 0.005 | 6.79E-05 | 80.4 |
| 60 | BIOCARTA_P27_PATHWAY | 0.005 | 6.39E-05 | 80.1 |
| 61 | SA_G2_AND_M_PHASES | 0.008 | 1.02E-04 | 80.0 |
| 62 | REACTOME_POLYMERASE_SWITCHING_ON_THE_C_STRAND_OF_THE_TELOMERE | 0.007 | 8.67E-05 | 79.5 |
| 63 | REACTOME_G2_M_DNA_DAMAGE_CHECKPOINT | 0.006 | 7.04E-05 | 79.1 |
| 64 | SA_REG_CASCADE_OF_CYCLIN_EXPR | 0.010 | 1.30E-04 | 78.9 |
| 65 | REACTOME_PROCESSING_OF_DNA_DOUBLE_STRAND_BREAK_ENDS | 0.005 | 6.88E-05 | 78.1 |
| 66 | BIOCARTA_BARD1_PATHWAY | 0.010 | 1.25E-04 | 77.3 |
| 67 | BIOCARTA_RB_PATHWAY | 0.008 | 1.02E-04 | 76.9 |
| 68 | REACTOME_PRC2_METHYLATES_HISTONES_AND_DNA | 0.006 | 7.33E-05 | 76.8 |
| 69 | REACTOME_APC_C_MEDIATED_DEGRADATION_OF_CELL_CYCLE_PROTEINS | 0.004 | 5.39E-05 | 76.8 |
| 70 | REACTOME_APC_CDC20_MEDIATED_DEGRADATION_OF_NEK2A | 0.005 | 6.33E-05 | 76.7 |
| 71 | REACTOME_REGULATION_OF_TP53_ACTIVITY_THROUGH_PHOSPHORYLATION | 0.004 | 5.51E-05 | 76.4 |
| 72 | REACTOME_RESOLUTION_OF_D_LOOP_STRUCTURES_THROUGH_SYNTHESIS_DEPENDENT_STRAND_ANNEALING_SDSA | 0.009 | 1.18E-04 | 76.2 |
| 73 | REACTOME_RESOLUTION_OF_D_LOOP_STRUCTURES | 0.008 | 1.04E-04 | 76.1 |
| 74 | REACTOME_HDR_THROUGH_HOMOLOGOUS_RECOMBINATION_HRR | 0.006 | 7.70E-05 | 75.7 |
| 75 | REACTOME_HOMOLOGOUS_DNA_PAIRING_AND_STRAND_EXCHANGE | 0.007 | 9.77E-05 | 75.7 |
| 76 | REACTOME_NUCLEAR_ENVELOPE_BREAKDOWN | 0.004 | 5.81E-05 | 75.6 |
| 77 | REACTOME_TELOMERE_C_STRAND_LAGGING_STRAND_SYNTHESIS | 0.006 | 8.50E-05 | 75.6 |
| 78 | BIOCARTA_CDC25_PATHWAY | 0.010 | 1.34E-04 | 74.9 |
| 79 | REACTOME_TELOMERE_MAINTENANCE | 0.006 | 7.48E-05 | 74.8 |
| 80 | REACTOME_EXTENSION_OF_TELOMERES | 0.006 | 7.48E-05 | 74.8 |
| 81 | PID_BARD1_PATHWAY | 0.005 | 7.05E-05 | 74.8 |
| 82 | REACTOME_AURKA_ACTIVATION_BY_TPX2 | 0.004 | 5.41E-05 | 74.7 |
| 83 | REACTOME_LAGGING_STRAND_SYNTHESIS | 0.006 | 8.56E-05 | 74.3 |
| 84 | PID_FANCONI_PATHWAY | 0.006 | 8.40E-05 | 74.2 |
| 85 | REACTOME_NUCLEAR_PORE_COMPLEX_NPC_DISASSEMBLY | 0.005 | 6.63E-05 | 74.2 |
| 86 | REACTOME_PROCESSIVE_SYNTHESIS_ON_THE_C_STRAND_OF_THE_TELOMERE | 0.006 | 7.68E-05 | 73.9 |
| 87 | REACTOME_POLYMERASE_SWITCHING | 0.007 | 8.81E-05 | 73.8 |
| 88 | REACTOME_SWITCHING_OF_ORIGINS_TO_A_POST_REPLICATIVE_STATE | 0.004 | 4.87E-05 | 73.8 |
| 89 | REACTOME_DNA_METHYLATION | 0.012 | 1.61E-04 | 73.5 |
| 90 | BIOCARTA_CELLCYCLE_PATHWAY | 0.007 | 8.86E-05 | 73.4 |
| 91 | BIOCARTA_SRCRPTP_PATHWAY | 0.007 | 9.98E-05 | 73.1 |
| 92 | REACTOME_ORC1_REMOVAL_FROM_CHROMATIN | 0.004 | 5.01E-05 | 72.8 |
| 93 | REACTOME_ASSEMBLY_OF_THE_PRE_REPLICATIVE_COMPLEX | 0.004 | 5.30E-05 | 72.6 |
| 94 | REACTOME_TRANSCRIPTIONAL_REGULATION_BY_E2F6 | 0.005 | 6.47E-05 | 72.5 |
| 95 | BIOCARTA_ATRBRCA_PATHWAY | 0.006 | 8.74E-05 | 72.4 |
| 96 | BIOCARTA_PLK3_PATHWAY | 0.006 | 8.53E-05 | 71.9 |
| 97 | REACTOME_NUCLEAR_ENVELOPE_NE_REASSEMBLY | 0.004 | 5.08E-05 | 71.9 |
| 98 | REACTOME_RECOGNITION_OF_DNA_DAMAGE_BY_PCNA_CONTAINING_REPLICATION_COMPLEX | 0.004 | 5.46E-05 | 71.6 |
| 99 | REACTOME_INHIBITION_OF_THE_PROTEOLYTIC_ACTIVITY_OF_APC_C_REQUIRED_FOR_THE_ONSET_OF_ANAPHASE_BY_MITOTIC_SPINDLE_CHECKPOINT_COMPONENTS | 0.005 | 6.86E-05 | 71.5 |
| 100 | REACTOME_PHOSPHORYLATION_OF_THE_APC_C | 0.005 | 6.80E-05 | 71.1 |
| 101 | BIOCARTA_G1_PATHWAY | 0.006 | 7.78E-05 | 70.9 |
| 102 | REACTOME_REGULATION_OF_TP53_ACTIVITY | 0.003 | 4.38E-05 | 70.4 |
| 103 | REACTOME_DNA_DAMAGE_TELOMERE_STRESS_INDUCED_SENESCENCE | 0.005 | 6.68E-05 | 70.4 |
| 104 | REACTOME_REGULATION_OF_PLK1_ACTIVITY_AT_G2_M_TRANSITION | 0.003 | 4.76E-05 | 70.1 |
| 105 | BIOCARTA_EFP_PATHWAY | 0.006 | 8.13E-05 | 70.1 |
| 106 | REACTOME_RESOLUTION_OF_AP_SITES_VIA_THE_MULTIPLE_NUCLEOTIDE_PATCH_REPLACEMENT_PATHWAY | 0.005 | 6.62E-05 | 69.8 |
| 107 | BIOCARTA_FBW7_PATHWAY | 0.005 | 6.87E-05 | 69.8 |
| 108 | REACTOME_HDR_THROUGH_SINGLE_STRAND_ANNEALING_SSA | 0.006 | 8.65E-05 | 69.7 |
| 109 | REACTOME_PCNA_DEPENDENT_LONG_PATCH_BASE_EXCISION_REPAIR | 0.005 | 7.18E-05 | 69.4 |
| 110 | REACTOME_PROCESSIVE_SYNTHESIS_ON_THE_LAGGING_STRAND | 0.006 | 8.53E-05 | 69.1 |
| 111 | REACTOME_DNA_REPLICATION_INITIATION | 0.007 | 1.07E-04 | 68.6 |
| 112 | REACTOME_G2_PHASE | 0.014 | 2.03E-04 | 68.5 |
| 113 | KEGG_MISMATCH_REPAIR | 0.005 | 7.73E-05 | 68.3 |
| 114 | REACTOME_DNA_REPAIR | 0.003 | 4.65E-05 | 68.2 |
| 115 | REACTOME_TRANSCRIPTIONAL_REGULATION_BY_TP53 | 0.002 | 3.40E-05 | 68.1 |
| 116 | REACTOME_APC_C_CDC20_MEDIATED_DEGRADATION_OF_CYCLIN_B | 0.004 | 5.69E-05 | 67.7 |
| 117 | REACTOME_CELLULAR_SENESCENCE | 0.003 | 3.96E-05 | 67.5 |
| 118 | REACTOME_ASSEMBLY_OF_THE_ORC_COMPLEX_AT_THE_ORIGIN_OF_REPLICATION | 0.008 | 1.18E-04 | 66.9 |
| 119 | REACTOME_GAP_FILLING_DNA_REPAIR_SYNTHESIS_AND_LIGATION_IN_GG_NER | 0.004 | 5.79E-05 | 66.5 |
| 120 | REACTOME_POSTMITOTIC_NUCLEAR_PORE_COMPLEX_NPC_REFORMATION | 0.004 | 6.12E-05 | 66.5 |
| 121 | REACTOME_INTERACTIONS_OF_REV_WITH_HOST_CELLULAR_PROTEINS | 0.004 | 5.90E-05 | 66.4 |
| 122 | REACTOME_KINESINS | 0.006 | 9.48E-05 | 66.2 |
| 123 | PID_ATM_PATHWAY | 0.004 | 6.57E-05 | 66.2 |
| 124 | REACTOME_MEIOTIC_RECOMBINATION | 0.007 | 1.00E-04 | 66.2 |
| 125 | REACTOME_MITOTIC_PROPHASE | 0.004 | 5.35E-05 | 65.9 |
| 126 | REACTOME_TFAP2A_ACTS_AS_A_TRANSCRIPTIONAL_REPRESSOR_DURING_RETINOIC_ACID_INDUCED_CELL_DIFFERENTIATION | 0.008 | 1.26E-04 | 65.8 |
| 127 | REACTOME_MISMATCH_REPAIR | 0.005 | 7.47E-05 | 65.8 |
| 128 | BIOCARTA_RAN_PATHWAY | 0.004 | 6.03E-05 | 65.8 |
| 129 | REACTOME_REMOVAL_OF_THE_FLAP_INTERMEDIATE_FROM_THE_C_STRAND | 0.005 | 6.90E-05 | 65.7 |
| 130 | REACTOME_MITOTIC_TELOPHASE_CYTOKINESIS | 0.007 | 1.10E-04 | 65.5 |
| 131 | REACTOME_INHIBITION_OF_REPLICATION_INITIATION_OF_DAMAGED_DNA_BY_RB1_E2F1 | 0.004 | 6.96E-05 | 64.1 |
| 132 | REACTOME_MASTL_FACILITATES_MITOTIC_PROGRESSION | 0.004 | 6.75E-05 | 64.1 |
| 133 | REACTOME_FANCONI_ANEMIA_PATHWAY | 0.005 | 7.09E-05 | 63.7 |
| 134 | BIOCARTA_SKP2E2F_PATHWAY | 0.007 | 1.07E-04 | 62.8 |
| 135 | REACTOME_CYCLIN_A_CDK2_ASSOCIATED_EVENTS_AT_S_PHASE_ENTRY | 0.003 | 4.00E-05 | 62.7 |
| 136 | REACTOME_NUCLEAR_IMPORT_OF_REV_PROTEIN | 0.004 | 6.22E-05 | 62.6 |
| 137 | REACTOME_COPI_DEPENDENT_GOLGI_TO_ER_RETROGRADE_TRAFFIC | 0.004 | 6.01E-05 | 62.3 |
| 138 | BIOCARTA_PTC1_PATHWAY | 0.008 | 1.24E-04 | 62.3 |
| 139 | REACTOME_TRANSPORT_OF_MATURE_MRNAS_DERIVED_FROM_INTRONLESS_TRANSCRIPTS | 0.003 | 5.64E-05 | 62.0 |
| 140 | REACTOME_NS1_MEDIATED_EFFECTS_ON_HOST_PATHWAYS | 0.004 | 5.77E-05 | 61.9 |
| 141 | REACTOME_VIRAL_MESSENGER_RNA_SYNTHESIS | 0.003 | 4.99E-05 | 61.3 |
| 142 | REACTOME_TRANSCRIPTIONAL_REGULATION_BY_SMALL_RNAS | 0.003 | 5.06E-05 | 60.6 |
| 143 | REACTOME_REGULATION_OF_GLUCOKINASE_BY_GLUCOKINASE_REGULATORY_PROTEIN | 0.004 | 6.55E-05 | 60.5 |
| 144 | REACTOME_SYNTHESIS_OF_ACTIVE_UBIQUITIN_ROLES_OF_E1_AND_E2_ENZYMES | 0.002 | 3.78E-05 | 60.3 |
| 145 | REACTOME_SUMOYLATION_OF_RNA_BINDING_PROTEINS | 0.003 | 5.31E-05 | 60.2 |
| 146 | REACTOME_RECRUITMENT_OF_MITOTIC_CENTROSOME_PROTEINS_AND_COMPLEXES | 0.003 | 4.93E-05 | 60.1 |
| 147 | BIOCARTA_NPC_PATHWAY | 0.004 | 5.91E-05 | 59.8 |
| 148 | REACTOME_EPIGENETIC_REGULATION_OF_GENE_EXPRESSION | 0.003 | 4.47E-05 | 59.7 |
| 149 | REACTOME_HDR_THROUGH_MMEJ_ALT_NHEJ | 0.005 | 8.97E-05 | 59.6 |
| 150 | REACTOME_EXPORT_OF_VIRAL_RIBONUCLEOPROTEINS_FROM_NUCLEUS | 0.004 | 6.29E-05 | 59.5 |
| 151 | REACTOME_TRNA_PROCESSING_IN_THE_NUCLEUS | 0.003 | 5.17E-05 | 59.3 |
| 152 | REACTOME_TRANSPORT_OF_MATURE_TRANSCRIPT_TO_CYTOPLASM | 0.003 | 4.49E-05 | 59.2 |
| 153 | REACTOME_TRANSPORT_OF_THE_SLBP_DEPENDANT_MATURE_MRNA | 0.004 | 6.19E-05 | 59.0 |
| 154 | REACTOME_APC_C_CDH1_MEDIATED_DEGRADATION_OF_CDC20_AND_OTHER_APC_C_CDH1_TARGETED_PROTEINS_IN_LATE_MITOSIS_EARLY_G1 | 0.003 | 4.75E-05 | 59.0 |
| 155 | REACTOME_SIGNALING_BY_RHO_GTPASES | 0.003 | 4.57E-05 | 58.8 |
| 156 | REACTOME_SUMOYLATION_OF_SUMOYLATION_PROTEINS | 0.004 | 6.02E-05 | 58.7 |
| 157 | REACTOME_SUMOYLATION_OF_DNA_DAMAGE_RESPONSE_AND_REPAIR_PROTEINS | 0.003 | 5.18E-05 | 58.7 |
| 158 | REACTOME_DNA_DAMAGE_BYPASS | 0.003 | 4.62E-05 | 57.7 |
| 159 | REACTOME_FORMATION_OF_SENESCENCE_ASSOCIATED_HETEROCHROMATIN_FOCI_SAHF | 0.005 | 8.45E-05 | 57.6 |
| 160 | REACTOME_RECRUITMENT_OF_NUMA_TO_MITOTIC_CENTROSOMES | 0.003 | 5.32E-05 | 57.4 |
| 161 | REACTOME_INTERACTIONS_OF_VPR_WITH_HOST_CELLULAR_PROTEINS | 0.003 | 5.84E-05 | 57.0 |
| 162 | REACTOME_BASE_EXCISION_REPAIR | 0.003 | 6.03E-05 | 56.9 |
| 163 | BIOCARTA_SAM68_PATHWAY | 0.004 | 6.52E-05 | 56.7 |
| 164 | REACTOME_GOLGI_TO_ER_RETROGRADE_TRANSPORT | 0.003 | 5.07E-05 | 56.5 |
| 165 | REACTOME_G1_S_DNA_DAMAGE_CHECKPOINTS | 0.003 | 4.49E-05 | 56.4 |
| 166 | REACTOME_TRANSLESION_SYNTHESIS_BY_POLH | 0.003 | 5.10E-05 | 56.3 |
| 167 | REACTOME_SNRNP_ASSEMBLY | 0.003 | 5.66E-05 | 56.3 |
| 168 | REACTOME_TRANSLESION_SYNTHESIS_BY_POLK | 0.003 | 5.49E-05 | 55.8 |
| 169 | REACTOME_SUMOYLATION_OF_UBIQUITINYLATION_PROTEINS | 0.003 | 5.72E-05 | 55.1 |
| 170 | REACTOME_HCMV_EARLY_EVENTS | 0.003 | 5.50E-05 | 54.9 |
| 171 | KEGG_OOCYTE_MEIOSIS | 0.003 | 5.19E-05 | 54.9 |
| 172 | REACTOME_CYCLIN_D_ASSOCIATED_EVENTS_IN_G1 | 0.003 | 4.73E-05 | 54.7 |
| 173 | REACTOME_TRANSLESION_SYNTHESIS_BY_Y_FAMILY_DNA_POLYMERASES_BYPASSES_LESIONS_ON_DNA_TEMPLATE | 0.002 | 4.53E-05 | 54.5 |
| 174 | PID_P73PATHWAY | 0.003 | 6.07E-05 | 54.1 |
| 175 | SA_G1_AND_S_PHASES | 0.004 | 8.00E-05 | 53.3 |
| 176 | REACTOME_TERMINATION_OF_TRANSLESION_DNA_SYNTHESIS | 0.003 | 4.90E-05 | 53.2 |
| 177 | REACTOME_RESOLUTION_OF_ABASIC_SITES_AP_SITES | 0.003 | 6.27E-05 | 53.1 |
| 178 | REACTOME_POLB_DEPENDENT_LONG_PATCH_BASE_EXCISION_REPAIR | 0.004 | 6.80E-05 | 53.1 |
| 179 | REACTOME_SCF_SKP2_MEDIATED_DEGRADATION_OF_P27_P21 | 0.002 | 4.42E-05 | 52.5 |
| 180 | REACTOME_ANCHORING_OF_THE_BASAL_BODY_TO_THE_PLASMA_MEMBRANE | 0.003 | 5.20E-05 | 52.3 |
| 181 | PID_RB_1PATHWAY | 0.003 | 5.62E-05 | 52.2 |
| 182 | REACTOME_CONVERSION_FROM_APC_C_CDC20_TO_APC_C_CDH1_IN_LATE_ANAPHASE | 0.003 | 5.48E-05 | 52.2 |
| 183 | REACTOME_SUMOYLATION_OF_CHROMATIN_ORGANIZATION_PROTEINS | 0.003 | 5.40E-05 | 52.0 |
| 184 | REACTOME_HCMV_LATE_EVENTS | 0.002 | 3.99E-05 | 52.0 |
| 185 | REACTOME_NUCLEOBASE_BIOSYNTHESIS | 0.004 | 7.22E-05 | 52.0 |
| 186 | REACTOME_TP53_REGULATES_TRANSCRIPTION_OF_DNA_REPAIR_GENES | 0.002 | 4.44E-05 | 51.7 |
| 187 | REACTOME_HCMV_INFECTION | 0.002 | 4.31E-05 | 51.1 |
| 188 | REACTOME_TELOMERE_EXTENSION_BY_TELOMERASE | 0.004 | 8.19E-05 | 51.1 |
| 189 | REACTOME_DEPOLYMERISATION_OF_THE_NUCLEAR_LAMINA | 0.004 | 7.18E-05 | 50.9 |
| 190 | REACTOME_THE_ROLE_OF_GTSE1_IN_G2_M_PROGRESSION_AFTER_G2_CHECKPOINT | 0.003 | 5.47E-05 | 50.5 |
| 191 | REACTOME_PURINE_RIBONUCLEOSIDE_MONOPHOSPHATE_BIOSYNTHESIS | 0.004 | 7.05E-05 | 50.3 |
| 192 | KEGG_P53_SIGNALING_PATHWAY | 0.003 | 6.15E-05 | 50.3 |
| 193 | REACTOME_ESTABLISHMENT_OF_SISTER_CHROMATID_COHESION | 0.006 | 1.19E-04 | 50.0 |
| 194 | REACTOME_PROCESSING_OF_CAPPED_INTRONLESS_PRE_MRNA | 0.002 | 4.28E-05 | 49.5 |
| 195 | REACTOME_SUMOYLATION_OF_DNA_METHYLATION_PROTEINS | 0.004 | 7.24E-05 | 49.4 |
| 196 | REACTOME_ACTIVATION_OF_NOXA_AND_TRANSLOCATION_TO_MITOCHONDRIA | 0.006 | 1.18E-04 | 49.4 |
| 197 | REACTOME_DUAL_INCISION_IN_GG_NER | 0.002 | 5.06E-05 | 48.4 |
| 198 | REACTOME_HIV_LIFE_CYCLE | 0.002 | 3.82E-05 | 48.0 |
| 199 | REACTOME_PROCESSING_OF_CAPPED_INTRON_CONTAINING_PRE_MRNA | 0.002 | 3.60E-05 | 47.9 |
| 200 | REACTOME_TRNA_PROCESSING | 0.002 | 5.24E-05 | 47.7 |

### Table S3. Potential m6A modification targets associated with the m6A signature

| Gene | Frequency* | Chr | Gene type | Gene ID | OncoScore |
| --- | --- | --- | --- | --- | --- |
| *BCL9L* | 17 | chr11 | protein_coding | ENSG00000186174 | 70.76 |
| *STK40* | 15 | chr1 | protein_coding | ENSG00000196182 | 27.09 |
| *LDB2* | 15 | chr4 | protein_coding | ENSG00000169744 | 23.50 |
| *SETD1B* | 14 | chr12 | protein_coding | ENSG00000139718 | 52.00 |
| *VMP1* | 14 | chr17 | protein_coding | ENSG00000062716 | 46.02 |
| *PRRC2A* | 14 | chr6 | protein_coding | ENSG00000204469 | 41.40 |
| *NUMA1* | 14 | chr11 | protein_coding | ENSG00000137497 | 40.02 |
| *LAG3* | 13 | chr12 | protein_coding | ENSG00000089692 | 56.20 |
| *SPEN* | 13 | chr1 | protein_coding | ENSG00000065526 | 36.40 |
| *RPRD2* | 13 | chr1 | protein_coding | ENSG00000163125 | 30.66 |
| *CD93* | 13 | chr20 | protein_coding | ENSG00000125810 | 29.22 |
| *ATN1* | 13 | chr12 | protein_coding | ENSG00000111676 | 13.42 |
| *CXorf36* | 13 | chrX | protein_coding | ENSG00000147113 | 11.39 |
| *ARID1A* | 12 | chr1 | protein_coding | ENSG00000117713 | 83.79 |
| *RNF31* | 12 | chr14 | protein_coding | ENSG00000092098 | 59.89 |
| *NKIRAS2* | 12 | chr17 | protein_coding | ENSG00000168256 | 48.93 |
| *ECSCR* | 12 | chr5 | protein_coding | ENSG00000249751 | 38.01 |
| *UBR4* | 12 | chr1 | protein_coding | ENSG00000127481 | 35.75 |
| *CDH5* | 12 | chr16 | protein_coding | ENSG00000179776 | 34.06 |
| *SPARC* | 12 | chr5 | protein_coding | ENSG00000113140 | 33.19 |
| *HCFC1* | 12 | chrX | protein_coding | ENSG00000172534 | 31.27 |
| *MEF2D* | 12 | chr1 | protein_coding | ENSG00000116604 | 30.32 |
| *ARAP3* | 12 | chr5 | protein_coding | ENSG00000120318 | 25.21 |
| *CRY2* | 12 | chr11 | protein_coding | ENSG00000121671 | 12.98 |
| *LRRC47* | 12 | chr1 | protein_coding | ENSG00000130764 | 0.00 |
| *RP11-389C8.2* | 12 | chr5 | sense_overlapping | ENSG00000261269 | 0.00 |
| *OCIAD2* | 11 | chr4 | protein_coding | ENSG00000145247 | 66.10 |
| *CDCA8* | 11 | chr1 | protein_coding | ENSG00000134690 | 61.70 |
| *HIGD1B* | 11 | chr17 | protein_coding | ENSG00000131097 | 56.93 |
| *TLN1* | 11 | chr9 | protein_coding | ENSG00000137076 | 48.52 |
| *BRD2* | 11 | chr6 | protein_coding | ENSG00000204256 | 48.20 |
| *ADGRL4* | 11 | chr1 | protein_coding | ENSG00000162618 | 44.43 |
| *TXLNA* | 11 | chr1 | protein_coding | ENSG00000084652 | 43.68 |
| *MAPKAPK2* | 11 | chr1 | protein_coding | ENSG00000162889 | 41.89 |
| *VPS25* | 11 | chr17 | protein_coding | ENSG00000131475 | 38.17 |
| *DPF2* | 11 | chr11 | protein_coding | ENSG00000133884 | 34.01 |
| *ROBO4* | 11 | chr11 | protein_coding | ENSG00000154133 | 33.26 |
| *BAHD1* | 11 | chr15 | protein_coding | ENSG00000140320 | 23.44 |
| *ARL15* | 11 | chr5 | protein_coding | ENSG00000185305 | 22.50 |
| *CSNK1G3* | 11 | chr5 | protein_coding | ENSG00000151292 | 20.44 |
| *NREP* | 11 | chr5 | protein_coding | ENSG00000134986 | 19.58 |
| *CIC* | 11 | chr19 | protein_coding | ENSG00000079432 | 19.21 |
| *NDUFAF2* | 11 | chr5 | protein_coding | ENSG00000164182 | 18.83 |
| *ATP5H* | 11 | chr17 | protein_coding | ENSG00000167863 | 17.47 |
| *ADAR* | 11 | chr1 | protein_coding | ENSG00000160710 | 16.61 |
| *PCDH12* | 11 | chr5 | protein_coding | ENSG00000113555 | 11.31 |
| *ELMOD2* | 11 | chr4 | protein_coding | ENSG00000179387 | 11.23 |
| *SLC25A14* | 11 | chrX | protein_coding | ENSG00000102078 | 10.90 |
| *OSTC* | 11 | chr4 | protein_coding | ENSG00000198856 | 9.92 |
| *CBR4* | 11 | chr4 | protein_coding | ENSG00000145439 | 2.98 |
| *TBC1D13* | 11 | chr9 | protein_coding | ENSG00000107021 | 0.00 |
| *RP11-257O5.2* | 10 | chr17 | TEC | ENSG00000279059 | NA |
| *NME1* | 10 | chr17 | protein_coding | ENSG00000239672 | 82.67 |
| *MYCT1* | 10 | chr6 | protein_coding | ENSG00000120279 | 73.56 |
| *YY1AP1* | 10 | chr1 | protein_coding | ENSG00000163374 | 71.59 |
| *PLXDC1* | 10 | chr17 | protein_coding | ENSG00000161381 | 65.91 |
| *COL18A1* | 10 | chr21 | protein_coding | ENSG00000182871 | 60.03 |
| *CMTM3* | 10 | chr16 | protein_coding | ENSG00000140931 | 59.80 |
| *AFAP1L1* | 10 | chr5 | protein_coding | ENSG00000157510 | 58.17 |
| *KDM2A* | 10 | chr11 | protein_coding | ENSG00000173120 | 56.17 |
| *THRAP3* | 10 | chr1 | protein_coding | ENSG00000054118 | 55.20 |
| *CD34* | 10 | chr1 | protein_coding | ENSG00000174059 | 54.22 |
| *NOVA2* | 10 | chr19 | protein_coding | ENSG00000104967 | 54.08 |
| *SETD1A* | 10 | chr16 | protein_coding | ENSG00000099381 | 51.61 |
| *POLR2A* | 10 | chr17 | protein_coding | ENSG00000181222 | 47.85 |
| *MRPL58* | 10 | chr17 | protein_coding | ENSG00000167862 | 47.81 |
| *TAGLN* | 10 | chr11 | protein_coding | ENSG00000149591 | 47.29 |
| *SNIP1* | 10 | chr1 | protein_coding | ENSG00000163877 | 46.41 |
| *PDGFRB* | 10 | chr5 | protein_coding | ENSG00000113721 | 44.82 |
| *TCF4* | 10 | chr18 | protein_coding | ENSG00000196628 | 44.66 |
| *BCL6B* | 10 | chr17 | protein_coding | ENSG00000161940 | 44.26 |
| *ELK3* | 10 | chr12 | protein_coding | ENSG00000111145 | 44.21 |
| *NOTCH4* | 10 | chr6 | protein_coding | ENSG00000204301 | 44.20 |
| *HYAL2* | 10 | chr3 | protein_coding | ENSG00000068001 | 43.92 |
| *BRPF3* | 10 | chr6 | protein_coding | ENSG00000096070 | 41.94 |
| *DCAF11* | 10 | chr14 | protein_coding | ENSG00000100897 | 40.88 |
| *KLHL21* | 10 | chr1 | protein_coding | ENSG00000162413 | 40.88 |
| *PRRC2B* | 10 | chr9 | protein_coding | ENSG00000130723 | 40.88 |
| *CRTC2* | 10 | chr1 | protein_coding | ENSG00000160741 | 40.07 |
| *C6orf106* | 10 | chr6 | protein_coding | ENSG00000196821 | 38.78 |
| *TECPR2* | 10 | chr14 | protein_coding | ENSG00000196663 | 37.66 |
| *POLR2J4* | 10 | chr7 | processed_transcript | ENSG00000214783 | 37.50 |
| *POLR2J4* | 10 | chr7 | transcribed_unprocessed_pseudogene | ENSG00000272655 | 37.50 |
| *ARHGEF15* | 10 | chr17 | protein_coding | ENSG00000198844 | 36.05 |
| *TIE1* | 10 | chr1 | protein_coding | ENSG00000066056 | 34.78 |
| *SEC14L1* | 10 | chr17 | protein_coding | ENSG00000129657 | 34.53 |
| *CCNH* | 10 | chr5 | protein_coding | ENSG00000134480 | 33.81 |
| *DVL1* | 10 | chr1 | protein_coding | ENSG00000107404 | 30.77 |
| *S1PR1* | 10 | chr1 | protein_coding | ENSG00000170989 | 30.60 |
| *F2R* | 10 | chr5 | protein_coding | ENSG00000181104 | 30.29 |
| *SCYL1* | 10 | chr11 | protein_coding | ENSG00000142186 | 30.13 |
| *FNBP1* | 10 | chr9 | protein_coding | ENSG00000187239 | 29.56 |
| *ACTA2* | 10 | chr10 | protein_coding | ENSG00000107796 | 28.82 |
| *CHD8* | 10 | chr14 | protein_coding | ENSG00000100888 | 25.95 |
| *CNN1* | 10 | chr19 | protein_coding | ENSG00000130176 | 25.87 |
| *PPP1R12B* | 10 | chr1 | protein_coding | ENSG00000077157 | 25.34 |
| *AP5B1* | 10 | chr11 | protein_coding | ENSG00000254470 | 21.07 |
| *EMCN* | 10 | chr4 | protein_coding | ENSG00000164035 | 20.53 |
| *ATG2A* | 10 | chr11 | protein_coding | ENSG00000110046 | 18.58 |
| *SF1* | 10 | chr11 | protein_coding | ENSG00000168066 | 18.48 |
| *MFN2* | 10 | chr1 | protein_coding | ENSG00000116688 | 14.65 |
| *PLEKHG1* | 10 | chr6 | protein_coding | ENSG00000120278 | 13.98 |
| *TPRG1L* | 10 | chr1 | protein_coding | ENSG00000158109 | 12.50 |
| *ZNF574* | 10 | chr19 | protein_coding | ENSG00000105732 | 12.30 |
| *RAVER1* | 10 | chr19 | protein_coding | ENSG00000161847 | 10.58 |
| *GCC1* | 10 | chr7 | protein_coding | ENSG00000179562 | 10.53 |
| *HSPB7* | 10 | chr1 | protein_coding | ENSG00000173641 | 9.70 |
| *SLC25A46* | 10 | chr5 | protein_coding | ENSG00000164209 | 9.20 |
| *KCNMB1* | 10 | chr5 | protein_coding | ENSG00000145936 | 4.91 |
| *CCDC97* | 10 | chr19 | protein_coding | ENSG00000142039 | 0.00 |
| *LRRC41* | 10 | chr1 | protein_coding | ENSG00000132128 | 0.00 |
| *TMEM44* | 10 | chr3 | protein_coding | ENSG00000145014 | 0.00 |
| *UBTD2* | 10 | chr5 | protein_coding | ENSG00000168246 | 0.00 |
| *ZNF777* | 10 | chr7 | protein_coding | ENSG00000196453 | 0.00 |

*indicates the number of cancer types that the target gene is associated with.

### Table S4. Independent validation of the potential m6A targets

| Gene* | Breast cancer^#^ | | Lung cancer | | Gastric cancer | | Ovarian cancer | | Liver cancer | | **Meta-analysis** | | |
| --- | --- | --- | --- | --- | --- | --- | --- | --- | --- | --- | --- | --- | --- |
|  | HR (95%CI) | *P* | HR (95%CI) | *P* | HR (95%CI) | *P* | HR (95%CI) | *P* | HR (95%CI) | *P* | HR (95%CI) | *P* | *FDR* |
| *STK40* | 1.31 (0.99-1.72) | 0.056 | 0.55 (0.46-0.65) | 1.30E-12 | 1.46 (1.18-1.82) | 0.00054 | 1.01 (0.83-1.24) | 0.91 | 1.17 (0.83-1.66) | 0.36 | 0.92 (0.83-1.02) | 9.99E-02 | 1.95E-01 |
| *LDB2* | 0.72 (0.59-0.87) | 0.00073 | 0.6 (0.53-0.68) | 2.30E-15 | 0.86 (0.71-1.04) | 0.13 | 1.18 (1.03-1.34) | 0.014 | 0.51 (0.36-0.73) | 0.00014 | 0.81 (0.75-0.87) | 5.29E-09 | **9.43E-08** |
| *SETD1B* | 0.89 (0.74-1.08) | 0.24 | 0.72 (0.63-0.82) | 4.90E-07 | 1.17 (0.97-1.42) | 0.11 | 0.95 (0.83-1.08) | 0.43 | 0.72 (0.51-1.02) | 0.067 | 0.88 (0.81-0.94) | 4.37E-04 | **2.03E-03** |
| *VMP1* | 1.1 (0.91-1.33) | 0.31 | 0.85 (0.75-0.97) | 1.20E-02 | 0.9 (0.76-1.07) | 0.23 | 0.96 (0.85-1.1） | 0.58 | NA | NA | 0.92 (0.84-1.01) | 6.64E-02 | 1.51E-01 |
| *PRRC2A* | NA | NA | NA | NA | NA | NA | NA | NA | NA | NA | NA | NA | NA |
| *NUMA1* | 0.74 (0.61-0.89) | 0.0018 | 0.7 (0.61-0.79) | 2.50E-08 | 0.94 (0.78-1.14) | 5.30E-01 | 0.9 (0.79-1.02) | 0.1 | 0.86 (0.61-1.22) | 0.39 | 0.8 (0.75-0.86) | 2.12E-09 | **4.53E-08** |
| *LAG3* | 0.73 (0.58-0.92) | 0.0079 | 1.06 (0.94-1.2) | 3.50E-01 | 0.74 (0.61-0.9) | 0.0019 | 1.02 (0.9-1.16) | 0.78 | 0.82 (0.58-1.16) | 0.26 | 0.94 (0.88-1.02) | 1.27E-01 | 2.23E-01 |
| *SPEN* | 0.7 (0.58-0.85) | 0.00023 | 0.75 (0.66-0.85) | 1.00E-05 | 1.32 (1.09-1.6) | 0.0043 | 1.19 (1.05-1.36) | 0.0075 | 1.04 (0.73-1.46) | 0.84 | 0.95 (0.88-1.02) | 1.38E-01 | 2.32E-01 |
| *RPRD2* | 0.62 (0.47-0.84) | 0.0014 | 0.57 (0.49-0.68) | 6.70E-11 | 0.83 (0.63-1.07) | 0.15 | 0.97 (0.79-1.18) | 0.73 | 1.07 (0.76-1.52) | 0.7 | 0.75 (0.68-0.83) | 7.85E-08 | **9.33E-07** |
| *CD93* | 0.84 (0.7-1.02) | 0.08 | 0.68 (0.6-0.78) | 5.10E-09 | 0.96 (0.79-1.16) | 0.69 | 1.09 (0.96-1.24) | 0.17 | 1.02 (0.72-1.44) | 0.91 | 0.89 (0.82-0.96) | 2.02E-03 | **9.02E-03** |
| *ATN1* | 0.78 (0.63-0.96) | 0.02 | 1.22 (1.07-1.38) | 2.00E-03 | 1.01 (0.84-1.23) | 0.88 | 1.01 (0.89-1.15) | 0.87 | 1.11 (0.79-1.58) | 0.54 | 1.05 (0.98-1.13) | 1.90E-01 | 2.95E-01 |
| *CXorf36* | 0.78 (0.59-1.03) | 0.075 | 0.87 (0.73-1.02) | 8.50E-02 | 0.84 (0.64-1.1) | 0.21 | 1.13 (0.92-1.38) | 0.24 | 0.81 (0.57-1.14) | 0.23 | 0.91 (0.82-1) | 5.30E-02 | 1.26E-01 |
| *ARID1A* | 0.67 (0.54-0.82) | 7.90E-05 | 0.72 (0.63-0.82) | 3.70E-07 | 1.12 (0.92-1.36) | 0.25 | 1.16 (1.02-1.32) | 0.027 | 1.1 (0.78-1.55) | 0.59 | 0.91 (0.85-0.98) | 1.53E-02 | **4.21E-02** |
| *RNF31* | 1.12 (0.92-1.36) | 0.27 | 0.83 (0.73-0.94) | 3.30E-03 | 1.23 (1.01-1.49) | 0.034 | 0.93 (0.82-1.06) | 0.27 | 1.01 (0.72-1.43) | 0.94 | 0.96 (0.89-1.03) | 2.77E-01 | 3.84E-01 |
| *NKIRAS2* | 1.35 (1.1-1.66) | 0.0038 | 1.01 (0.89-1.15) | 8.40E-01 | 1.08 (0.89-1.3) | 0.45 | 1.03 (0.9-1.17) | 0.67 | 1.31 (0.92-1.86) | 0.13 | 1.08 (1-1.16) | 4.36E-02 | 1.06E-01 |
| *ECSCR* | 1.1 (0.84-1.45) | 0.47 | 0.67 (0.57-0.8) | 3.20E-06 | 0.97 (0.74-1.26) | 0.8 | 1.2 (0.98-1.47) | 0.079 | 0.58 (0.41-0.83) | 0.0024 | 0.88 (0.79-0.98) | 1.62E-02 | **4.32E-02** |
| *UBR4* | 0.85 (0.7-1.02) | 0.084 | 0.81 (0.72-0.92) | 1.00E-03 | 1.31 (1.08-1.58) | 0.0066 | 1.21 (1.07-1.38) | 0.0032 | 1.08 (0.77-1.53) | 0.65 | 1.01 (0.94-1.08) | 8.33E-01 | 8.93E-01 |
| *CDH5* | 0.79 (0.66-0.96) | 0.017 | 0.69 (0.61-0.78) | 9.50E-09 | 1.08 (0.89-1.31) | 0.44 | 1.01 (0.89-1.15) | 0.91 | 0.67 (0.47-0.94) | 0.022 | 0.84 (0.79-0.91) | 5.66E-06 | **3.76E-05** |
| *SPARC* | 0.75 (0.61-0.93) | 0.009 | 0.7 (0.62-0.79) | 2.90E-08 | 0.96 (0.79-1.16) | 0.66 | 1.24 (1.09-1.41) | 0.0011 | 0.83 (0.59-1.18) | 0.3 | 0.9 (0.83-0.97) | 3.73E-03 | **1.48E-02** |
| *HCFC1* | 0.85 (0.7-1.03) | 0.092 | 1.23 (1.08-1.39) | 1.60E-03 | 1.27 (1.05-1.54) | 0.015 | 0.96 (0.84-1.09) | 0.51 | 1.63 (1.15-2.31) | 0.0058 | 1.1 (1.02-1.18) | 1.39E-02 | **4.04E-02** |
| *MEF2D* | 0.7 (0.53-0.91) | 0.0089 | 0.53 (0.45-0.63) | 1.60E-13 | 1.06 (0.87-1.39) | 0.65 | 1.11 (0.91-1.36) | 0.3 | 0.96 (0.68-1.35) | 0.8 | 0.78 (0.7-0.86) | 2.23E-06 | **1.70E-05** |
| *ARAP3* | 0.89 (0.73-1.09) | 0.26 | 1 (0.88-1.14) | 9.70E-01 | 1.32 (1.09-1.6) | 0.0051 | 0.88 (0.77-1) | 0.048 | 0.78 (0.55-1.11) | 0.17 | 0.97 (0.9-1.05) | 4.50E-01 | 5.54E-01 |
| *CRY2* | 0.73 (0.6-0.89) | 0.0017 | 0.62 (0.54-0.7) | 5.30E-14 | 1.2 (0.99-1.46) | 0.061 | 1.05 (0.92-1.19) | 0.48 | 0.44 (0.31-0.63) | 4.60E-06 | 0.82 (0.76-0.88) | 3.21E-08 | **4.29E-07** |
| *LRRC47* | 0.76 (0.63-0.92) | 0.005 | 0.7 (0.61-0.79) | 2.20E-08 | 1.17 (0.96-1.41) | 0.12 | 1.17 (1.03-1.33) | 0.015 | 0.96 (0.68-1.36) | 0.84 | 0.91 (0.85-0.98) | 1.05E-02 | **3.40E-02** |
| *OCIAD2* | 1.25 (0.95-1.64) | 0.11 | 0.83 (0.71-0.98) | 2.80E-02 | 1.07 (0.82-1.4) | 0.63 | 0.84 (0.69-1.03) | 0.1 | 0.82 (0.58-1.15) | 0.25 | 0.91 (0.83-1.01) | 8.19E-02 | 1.68E-01 |
| *CDCA8* | 1.94 (1.56-2.42) | 1.70E-09 | 1.68 (1.48-1.92) | 9.00E-16 | 1.21 (1-1.47) | 0.048 | 1.06 (0.93-1.2) | 0.4 | 2.18 (1.53-3.12) | 1.20E-05 | 1.39 (1.29-1.5) | 1.78E-17 | **1.90E-15** |
| *HIGD1B* | 0.85 (0.69-1.06) | 0.15 | 0.73 (0.64-0.83) | 9.60E-07 | 1.33 (1.1-1.62) | 0.0033 | 1.07 (0.94-1.22) | 0.28 | 0.94 (0.66-1.32) | 0.72 | 0.93 (0.87-1.01) | 8.17E-02 | 1.68E-01 |
| *TLN1* | 0.74 (0.61-0.91) | 0.0038 | 1.18 (1.04-1.34) | 8.40E-03 | 0.88 (0.73-1.07) | 0.2 | 1.05 (0.93-1.2) | 0.42 | 0.9 (0.64-1.27) | 0.54 | 1.01 (0.94-1.09) | 8.02E-01 | 8.75E-01 |
| *BRD2* | 1.24 (1.03-1.5) | 0.024 | 0.99 (0.87-1.13) | 9.00E-01 | 1.01 (0.83-1.22) | 0.95 | 0.82 (0.72-0.93) | 0.0023 | 0.97 (0.68-1.37) | 0.84 | 0.96 (0.89-1.04) | 3.03E-01 | 4.10E-01 |
| *ADGRL4* | NA | NA | NA | NA | NA | NA | NA | NA | NA | NA | NA | NA | NA |
| *TXLNA* | 0.66 (0.54-0.8) | 3.30E-05 | 0.98 (0.87-1.12) | 8.10E-01 | 1.04 (0.86-1.26) | 0.71 | 1.06 (0.93-1.21) | 0.35 | 1.53 (1.08-2.18) | 0.016 | 0.97 (0.9-1.05) | 4.98E-01 | 6.06E-01 |
| *MAPKAPK2* | 0.82 (0.67-0.99) | 0.04 | 0.97 (0.86-1.1) | 6.60E-01 | 0.91 (0.75-1.11) | 0.35 | 0.99 (0.87-1.13) | 0.91 | 1.28 (0.91-1.81) | 0.16 | 0.95 (0.89-1.03) | 2.21E-01 | 3.16E-01 |
| *VPS25* | 0.77 (0.58-1.02) | 0.067 | 1.23 (1.04-1.45) | 1.30E-02 | 1.07 (0.82-1.4) | 0.61 | 1.1 (0.9-1.35) | 0.35 | 0.98 (0.7-1.39) | 0.93 | 1.08 (0.98-1.2) | 1.39E-01 | 2.32E-01 |
| *DPF2* | 0.7 (0.58-0.84) | 0.00017 | 0.93 (0.82-1.06) | 2.90E-01 | 1.11 (0.92-1.34) | 0.29 | 1.02 (0.9-1.16) | 0.76 | 1.29 (0.91-1.82) | 0.15 | 0.95 (0.89-1.03) | 2.12E-01 | 3.08E-01 |
| *ROBO4* | 1.87 (1.32-2.65) | 0.00038 | 0.63 (0.53-0.75) | 7.70E-08 | 0.96 (0.74-1.26) | 0.78 | 1.09 (0.89-1.34) | 0.38 | 0.48 (0.34-0.69) | 4.70E-05 | 0.85 (0.76-0.94) | 2.73E-03 | **1.17E-02** |
| *BAHD1* | 0.8 (0.66-0.97) | 0.02 | 1.24 (1.1-1.41) | 7.10E-04 | 1.19 (0.98-1.45) | 0.072 | 0.87 (0.77-0.99） | 0.038 | 0.67 (0.47-0.96) | 0.027 | 1.07 (0.98-1.17) | 1.35E-01 | 2.32E-01 |
| *ARL15* | 0.83 (0.69-1) | 0.052 | 0.86 (0.75-0.97) | 1.50E-02 | 0.91 (0.75-1.1) | 0.35 | 1.03 (0.9-1.17) | 0.71 | 0.83 (0.58-1.17) | 0.28 | 0.91 (0.85-0.98) | 1.09E-02 | **3.44E-02** |
| *CSNK1G3* | 0.61 (0.46-0.82) | 0.00092 | 0.67 (0.57-0.79) | 2.50E-06 | 1.1 (0.84-1.44) | 0.48 | 0.99 (0.81-1.21) | 0.9 | 1.04 (0.74-1.47) | 0.81 | 0.82 (0.74-0.91) | 1.35E-04 | **6.55E-04** |
| *NREP* | 1.42 (1.07-1.88) | 0.014 | 0.91 (0.77-1.07) | 2.60E-01 | 1 (0.77-1.31) | 0.99 | 0.88 (0.72-1.07) | 0.2 | NA | NA | 0.97 (0.88-1.08) | 6.09E-01 | 7.08E-01 |
| *CIC* | 0.8 (0.64-1.01) | 0.06 | 1.27 (1.12-1.44) | 2.30E-04 | 0.89 (0.74-1.08) | 0.25 | 1.06 (0.94-1.21) | 0.35 | 0.92 (0.65-1.3) | 0.65 | 1.06 (0.98-1.15) | 1.17E-01 | 2.12E-01 |
| *NDUFAF2* | 1.9 (1.45-2.49) | 2.70E-06 | 0.96 (0.82-1.14) | 6.70E-01 | 1.09 (0.83-1.42) | 0.55 | 0.78 (0.64-0.96) | 0.017 | 1.29 (0.91-1.82) | 0.15 | 1.06 (0.95-1.17) | 3.09E-01 | 4.14E-01 |
| *ATP5H* | 0.77 (0.62-0.94) | 0.012 | 1.37 (1.2-1.56) | 1.90E-06 | 1.18 (0.97-1.43) | 0.093 | 0.93 (0.82-1.06) | 0.27 | 0.8 (0.56-1.13) | 0.21 | 1.06 (0.98-1.14) | 1.24E-01 | 2.22E-01 |
| *ADAR* | 0.86 (0.7-1.06) | 0.15 | 0.92 (0.82-1.05) | 2.20E-01 | 1.03 (0.85-1.24) | 0.79 | 0.96 (0.85-1.09) | 0.55 | 1.13 (0.8-1.59) | 0.5 | 0.95 (0.88-1.03) | 1.96E-01 | 2.95E-01 |
| *PCDH12* | 1.41 (1.16-1.7) | 0.00039 | 0.87 (0.76-0.98) | 2.80E-02 | 1.11 (0.92-1.35) | 0.27 | 1.12 (0.99-1.28) | 0.08 | 0.75 (0.53-1.07) | 0.11 | 1.04 (0.96-1.11) | 3.33E-01 | 4.34E-01 |
| *ELMOD2* | 0.7 (0.52-0.94) | 0.018 | 0.97 (0.82-1.15) | 7.30E-01 | 0.9 (0.69-1.17) | 0.43 | 0.97 (0.79-1.18) | 0.74 | 1.14 (0.81-1.61) | 0.46 | 0.94 (0.84-1.04) | 1.99E-01 | 2.96E-01 |
| *SLC25A14* | 1.12 (0.91-1.38) | 0.29 | 0.76 (0.67-0.86) | 1.90E-05 | 1.42 (1.17-1.72) | 0.00033 | 0.9 (0.79-1.02) | 0.11 | 1.13 (0.8-1.59) | 0.49 | 0.94 (0.87-1.01) | 1.00E-01 | 1.95E-01 |
| *OSTC* | NA | NA | NA | NA | NA | NA | NA | NA | 1.01 (0.71-1.42) | 0.96 | 1.01 (0.72-1.42) | 9.54E-01 | 9.70E-01 |
| *CBR4* | 0.75 (0.61-0.92) | 0.0053 | 0.7 (0.62-0.79) | 2.20E-08 | 0.89 (0.73-1.08) | 0.23 | 0.87 (0.77-0.99) | 0.036 | 0.59 (0.42-0.84) | 0.0026 | 0.78 (0.72-0.84) | 1.68E-11 | **4.50E-10** |
| *TBC1D13* | 0.847 (0.71-1.05) | 0.14 | 1.07 (0.95-1.22) | 2.70E-01 | 0.93 (0.77-1.13) | 0.48 | 0.95 (0.84-1.08) | 0.45 | 1.25 (0.88-1.76) | 0.21 | 0.98 (0.91-1.06) | 6.80E-01 | 7.65E-01 |
| *NME1* | 1.33 (1.1-1.6) | 0.0031 | 1.65 (1.45-1.87) | 1.50E-14 | 1.38 (1.14-1.68) | 0.00092 | 1.05 (0.92-1.19) | 0.49 | 1.24 (0.88-1.75) | 0.23 | 1.32 (1.23-1.42) | 3.14E-14 | **1.68E-12** |
| *MYCT1* | 0.68 (0.5-0.92) | 0.011 | 0.76 (0.65-0.9) | 1.20E-03 | 1.02 (0.78-1.33) | 0.88 | 0.99 (0.81-1.22) | 0.95 | 0.51 (0.36-0.72) | 0.00013 | 0.81 (0.73-0.9) | 6.81E-05 | **3.64E-04** |
| *YY1AP1* | 0.72 (0.59-0.88) | 0.00095 | 0.93 (0.82-1.05) | 2.50E-01 | 0.94 (0.78-1.14) | 0.55 | 0.93 (0.82-1.06) | 0.27 | 1.26 (0.89-1.79) | 0.19 | 0.91 (0.85-0.98) | 1.40E-02 | **4.04E-02** |
| *PLXDC1* | 0.82 (0.68-1) | 0.05 | 0.77 (0.68-0.87) | 4.00E-05 | 1.07 (0.88-1.3) | 0.48 | 1.26 (1.11-1.43) | 0.00041 | 1.24 (0.87-1.75) | 0.23 | 0.98 (0.91-1.05) | 5.18E-01 | 6.16E-01 |
| *COL18A1* | 1.25 (1.04-1.51) | 0.02 | 1 (0.88-1.14) | 9.60E-01 | 1.23 (1.01-1.49) | 0.038 | 1.02 (0.9-1.16) | 0.73 | 0.61 (0.43-0.87) | 0.0052 | 1.05 (0.98-1.13) | 1.83E-01 | 2.88E-01 |
| *CMTM3* | 1.1 (0.84-1.45) | 0.49 | 1.02 (0.86-1.2) | 8.40E-01 | 1.31 (1-1.72) | 0.046 | 1.26 (1.03-1.55) | 0.024 | 1.01 (0.71-1.42) | 0.97 | 1.12 (1.01-1.24) | 2.65E-02 | 6.92E-02 |
| *AFAP1L1* | 1.33 (0.99-1.77) | 0.055 | 0.87 (0.74-1.02) | 8.90E-02 | 0.98 (0.75-1.29) | 0.9 | 1.01 (0.82-1.23) | 0.94 | 1.05 (0.75-1.49) | 0.77 | 0.98 (0.89-1.09) | 7.49E-01 | 8.34E-01 |
| *KDM2A* | 1.22 (0.99-1.5) | 0.057 | 0.71 (0.62-0.8) | 6.10E-08 | 1.31 (1.08-1.58) | 0.0065 | 1.01 (0.89-1.15) | 0.91 | 0.84 (0.6-1.19) | 0.33 | 0.94 (0.87-1.01) | 7.42E-02 | 1.62E-01 |
| *THRAP3* | 0.63 (0.47-0.83) | 0.001 | 1.57 (1.33-1.85) | 1.10E-07 | 1.06 (0.81-1.39) | 0.65 | 1.14 (0.93-1.39) | 0.21 | 0.95 (0.67-1.34) | 0.77 | 1.16 (1.05-1.28) | 4.42E-03 | **1.69E-02** |
| *CD34* | 0.73 (0.59-0.9) | 0.0037 | 0.98 (0.86-1.11) | 7.40E-01 | 0.92 (0.76-1.12) | 0.42 | 0.95 (0.84-1.08) | 0.47 | 0.65 (0.46-0.92) | 0.015 | 0.91 (0.84-0.98) | 1.19E-02 | **3.63E-02** |
| *NOVA2* | 1.22 (0.92-1.61) | 0.17 | 0.85 (0.72-1.01) | 6.00E-02 | 1.1 (0.84-1.43) | 0.5 | 1.09 (0.89-1.33) | 0.41 | 0.81 (0.58-1.15) | 0.24 | 0.99 (0.89-1.1) | 8.35E-01 | 8.93E-01 |
| *SETD1A* | 0.85 (0.69-1.05) | 0.13 | 1.27 (1.12-1.44) | 2.20E-04 | 1.17 (0.96-1.42) | 0.11 | 1.08 (0.95-1.23) | 0.25 | 0.95 (0.68-1.35) | 0.79 | 1.12 (1.04-1.2) | 3.74E-03 | **1.48E-02** |
| *POLR2A* | 0.78 (0.63-0.96) | 0.021 | 1.23 (1.09-1.4) | 1.20E-03 | 1.12 (0.92-1.36) | 0.24 | 0.97 (0.86-1.11) | 0.68 | 0.94 (0.67-1.33) | 0.73 | 1.04 (0.97-1.13) | 2.69E-01 | 3.79E-01 |
| *MRPL58* | NA | NA | NA | NA | NA | NA | NA | NA | NA | NA | NA | NA | NA |
| *TAGLN* | 0.92 (0.76-1.11) | 0.38 | 0.9 (0.8-1.03) | 1.20E-01 | 0.94 (0.78-1.14) | 0.52 | 1.16 (1.02-1.32) | 0.026 | 0.65 (0.46-0.92) | 0.016 | 0.98 (0.9-1.05) | 5.09E-01 | 6.12E-01 |
| *SNIP1* | 0.63 (0.48-0.83) | 8.00E-04 | 0.99 (0.84-1.17) | 9.00E-01 | 1.01 (0.77-1.32) | 0.94 | 1.07 (0.87-1.31) | 0.52 | 1.07 (0.76-1.51) | 0.69 | 0.96 (0.87-1.06) | 4.14E-01 | 5.21E-01 |
| *PDGFRB* | 0.84 (0.68-1.02) | 0.08 | 0.81 (0.71-0.92) | 8.80E-04 | 1.01 (0.84-1.23) | 0.89 | 1.22 (1.08-1.39) | 0.002 | 0.83 (0.59-1.17) | 0.28 | 0.96 (0.89-1.03) | 2.90E-01 | 3.98E-01 |
| *TCF4* | 0.74 (0.6-0.91) | 0.0037 | 0.86 (0.76-0.97) | 1.80E-02 | 1.02 (0.84-1.24) | 0.84 | 1.1 (0.97-1.26) | 0.13 | 0.86 (0.61-1.21) | 0.38 | 0.93 (0.86-1) | 5.74E-02 | 1.34E-01 |
| *BCL6B* | 1.55 (1.13-2.14) | 0.0066 | 0.81 (0.69-0.96) | 1.20E-02 | 0.99 (0.76-1.3) | 0.95 | 1.37 (1.12-1.68) | 0.0022 | 0.7 (0.5-0.99) | 0.045 | 1.02 (0.91-1.13) | 7.77E-01 | 8.57E-01 |
| *ELK3* | 0.79 (0.65-0.95) | 0.014 | 0.8 (0.7-0.9) | 4.00E-04 | 0.96 (0.79-1.17) | 0.7 | 1.18 (1.03-1.34) | 0.014 | 0.79 (0.56-1.12) | 0.19 | 0.92 (0.86-0.99) | 2.78E-02 | 7.09E-02 |
| *NOTCH4* | 0.99 (0.82-1.19) | 0.89 | 1.02 (0.9-1.16) | 7.40E-01 | 1.15 (0.95-1.39) | 0.16 | 0.92 (0.81-1.05) | 0.2 | 0.64 (0.45-0.9) | 0.01 | 0.98 (0.91-1.05) | 5.80E-01 | 6.82E-01 |
| *HYAL2* | 1.05 (0.87-1.27) | 0.61 | 0.9 (0.79-1.02) | 1.00E-01 | 0.78 (0.65-0.95) | 0.013 | 0.9 (0.79-1.02) | 0.099 | 0.85 (0.6-1.2) | 0.36 | 0.9 (0.84-0.97) | 4.68E-03 | **1.73E-02** |
| *BRPF3* | 0.76 (0.58-1) | 0.046 | 0.72 (0.61-0.85) | 8.40E-05 | 1 (0.77-1.31) | 0.98 | 1.17 (0.96-1.43) | 0.13 | 0.94 (0.66-1.32) | 0.71 | 0.88 (0.8-0.98) | 1.50E-02 | **4.21E-02** |
| *DCAF11* | 0.81 (0.67-0.98) | 0.027 | 1.02 (0.9-1.16) | 7.40E-01 | 1.03 (0.85-1.25) | 0.77 | 0.94 (0.82-1.06) | 0.31 | 0.65 (0.46-0.93) | 0.016 | 0.94 (0.88-1.01) | 1.05E-01 | 1.97E-01 |
| *KLHL21* | 0.89 (0.74-1.07) | 0.21 | 0.69 (0.61-0.79) | 1.50E-08 | 1.46 (1.21-1.77) | 0.00011 | 1.04 (0.92-1.18) | 0.54 | 1.14 (0.81-1.61) | 0.45 | 0.95 (0.88-1.02) | 1.49E-01 | 2.46E-01 |
| *PRRC2B* | 0.99 (0.75-1.29) | 0.92 | 1.18 (1-1.39) | 4.50E-02 | 0.92 (0.7-1.2) | 0.54 | 0.97 (0.79-1.19) | 0.77 | NA | NA | 1.05 (0.94-1.16) | 3.94E-01 | 5.02E-01 |
| *CRTC2* | 0.85 (0.65-1.12) | 0.24 | 1.36 (1.15-1.6) | 2.60E-04 | 0.96 (0.74-1.26) | 0.79 | 1.54 (1.25-1.89) | 3.40E-05 | 1.26 (0.89-1.78) | 0.19 | 1.24 (1.12-1.38) | 2.43E-05 | **1.44E-04** |
| *C6orf106* | 1.26 (1.04-1.52) | 0.017 | 0.68 (0.6-0.77) | 2.10E-09 | 1.54 (1.27-1.87) | 9.20E-06 | 0.99 (0.87-1.13) | 0.92 | 0.79 (0.56-1.11) | 0.17 | 0.95 (0.88-1.02) | 1.69E-01 | 2.70E-01 |
| *TECPR2* | 0.88 (0.73-1.06) | 0.18 | 0.6 (0.53-0.68) | 2.40E-15 | 1.23 (1.01-1.49) | 0.035 | 0.91 (0.8-1.03) | 0.14 | 1.19 (0.84-1.68) | 0.32 | 0.83 (0.78-0.9) | 6.31E-07 | **5.63E-06** |
| *ARHGEF15* | 0.86 (0.72-1.04) | 0.13 | 1.02 (0.9-1.16) | 7.30E-01 | 1.02 (0.85-1.24) | 0.81 | 0.88 (0.77-1) | 0.054 | 0.62 (0.44-0.88) | 0.0065 | 0.93 (0.86-1) | 4.03E-02 | 1.00E-01 |
| *TIE1* | 0.88 (0.73-1.06) | 0.17 | 0.93 (0.82-1.06) | 2.60E-01 | 0.99 (0.82-1.2) | 0.93 | 1.23 (1.08-1.4) | 0.0015 | 0.63 (0.45-0.9) | 0.0091 | 1 (0.93-1.08) | 9.40E-01 | 9.67E-01 |
| *SEC14L1* | 0.94 (0.78-1.14) | 0.55 | 0.81 (0.71-0.92) | 9.30E-04 | 0.88 (0.73-1.07) | 0.19 | 0.89 (0.78-1.01) | 0.063 | 0.8 (0.57-1.13) | 0.21 | 0.86 (0.8-0.93) | 9.34E-05 | **4.76E-04** |
| *CCNH* | 0.74 (0.61-0.89) | 0.0016 | 0.96 (0.84-1.08) | 4.80E-01 | 0.88 (0.72-1.06) | 0.17 | 1.11 (0.98-1.27) | 0.099 | 0.82 (0.58-1.16) | 0.27 | 0.94 (0.88-1.01) | 1.10E-01 | 2.02E-01 |
| *DVL1* | 1.13 (0.93-1.36) | 0.22 | 0.97 (0.86-1.1) | 6.50E-01 | 1.24 (1.02-1.5) | 0.029 | 1.02 (0.9-1.16) | 0.74 | 1.14 (0.81-1.61) | 0.46 | 1.05 (0.98-1.13) | 1.59E-01 | 2.58E-01 |
| *S1PR1* | 0.92 (0.76-1.11) | 0.39 | 0.7 (0.62-0.8) | 8.00E-08 | 0.78 (0.64-0.94) | 0.01 | 1.04 (0.92-1.19) | 0.51 | 0.5 (0.35-0.72) | 0.00012 | 0.83 (0.77-0.9) | 1.49E-06 | **1.22E-05** |
| *F2R* | 0.92 (0.76-1.11) | 0.36 | 0.84 (0.74-0.96) | 8.60E-03 | 1.16 (0.96-1.41) | 0.12 | 1.16 (1.02-1.32) | 0.025 | 0.85 (0.6-1.19) | 0.34 | 1 (0.92-1.07) | 9.08E-01 | 9.43E-01 |
| *SCYL1* | 1.16 (0.88-1.52) | 0.29 | 0.77 (0.66-0.91) | 2.30E-03 | 1.25 (0.96-1.63) | 0.1 | 1.02 (0.83-1.24) | 0.88 | 0.71 (0.5-1) | 0.051 | 0.94 (0.85-1.03) | 1.95E-01 | 2.95E-01 |
| *FNBP1* | 0.72 (0.6-0.87) | 0.00071 | 0.66 (0.58-0.75) | 1.80E-10 | 0.79 (0.65-0.96) | 0.016 | 0.88 (0.77-1) | 0.05 | 1.04 (0.74-1.47) | 0.83 | 0.77 (0.72-0.83) | 3.39E-12 | **1.21E-10** |
| *ACTA2* | 0.98 (0.81-1.18) | 0.82 | 0.74 (0.65-0.84) | 2.00E-06 | 0.93 (0.77-1.13) | 0.45 | 1.13 (1-1.29) | 0.058 | 0.67 (0.47-0.94) | 0.021 | 0.91 (0.84-0.98) | 1.01E-02 | **3.39E-02** |
| *CHD8* | 0.76 (0.63-0.92) | 0.0045 | 1.31 (1.16-1.49) | 2.20E-05 | 1.05 (0.87-1.27) | 0.62 | 0.89 (0.78-1.01) | 0.066 | 1.04 (0.74-1.47) | 0.83 | 1.02 (0.95-1.09) | 6.50E-01 | 7.39E-01 |
| *CNN1* | 0.83 (0.69-1) | 0.048 | 0.9 (0.79-1.02) | 8.50E-02 | 0.82 (0.68-0.99) | 0.041 | 1.13 (0.99-1.28) | 0.065 | 0.7 (0.5-0.99) | 0.044 | 0.94 (0.87-1.01) | 7.16E-02 | 1.60E-01 |
| *PPP1R12B* | 0.72 (0.6-0.88) | 0.00081 | 0.74 (0.66-0.85) | 5.40E-06 | 0.84 (0.69-1.01) | 0.067 | 0.96 (0.84-1.09) | 0.52 | 0.56 (0.4-0.8) | 0.0011 | 0.81 (0.76-0.88) | 1.01E-07 | **1.09E-06** |
| *AP5B1* | 0.81 (0.62-1.07) | 0.13 | 1.16 (0.99-1.37) | 7.20E-02 | 1.07 (0.82-1.4) | 0.6 | 1.02 (0.83-1.25) | 0.84 | NA | NA | 1.05 (0.94-1.17) | 3.94E-01 | 5.02E-01 |
| *EMCN* | 0.83 (0.63-1.08) | 0.17 | 0.51 (0.43-0.61) | 4.20E-15 | 1.01 (0.77-1.32) | 0.96 | 1.31 (1.07-1.6) | 0.0094 | 0.47 (0.33-0.67) | 1.90E-05 | 0.78 (0.7-0.87) | 3.37E-06 | **2.40E-05** |
| *ATG2A* | 0.92 (0.76-1.11) | 0.37 | 1.08 (0.95-1.22) | 2.60E-01 | 1.18 (0.97-1.43) | 0.093 | 0.97 (0.85-1.1) | 0.66 | 1 (0.71-1.42) | 0.99 | 1.03 (0.96-1.1) | 4.50E-01 | 5.54E-01 |
| *SF1* | 0.84 (0.7-1.02) | 0.073 | 0.86 (0.75-0.97) | 1.70E-02 | 1.29 (1.06-1.56) | 0.0099 | 1 (0.88-1.13) | 0.96 | 0.89 (0.63-1.26) | 0.53 | 0.96 (0.89-1.03) | 2.13E-01 | 3.08E-01 |
| *MFN2* | 0.83 (0.69-1.01) | 0.057 | 0.81 (0.71-0.92) | 1.20E-03 | 1.1 (0.91-1.33) | 0.34 | 1.18 (1.04-1.34) | 0.011 | 0.79 (0.56-1.11) | 0.17 | 0.96 (0.89-1.04) | 3.15E-01 | 4.16E-01 |
| *PLEKHG1* | 0.97 (0.74-1.27) | 0.84 | 0.68 (0.58-0.8) | 4.70E-06 | 1.2 (0.92-1.56) | 0.19 | 1.27 (1.04-1.55) | 0.021 | 0.73 (0.52-1.03) | 0.073 | 0.91 (0.83-1.01) | 7.72E-02 | 1.65E-01 |
| *TPRG1L* | 0.76 (0.58-0.99) | 0.043 | 0.56 (0.48-0.67) | 1.50E-11 | 0.95 (0.73-1.24) | 0.72 | 0.92 (0.75-1.13) | 0.42 | 0.96 (0.68-1.36) | 0.83 | 0.76 (0.69-0.84) | 3.01E-07 | **2.93E-06** |
| *ZNF574* | 1.09 (0.9-1.32) | 0.36 | 1.02 (0.9-1.16) | 7.70E-01 | 0.96 (0.79-1.16) | 0.65 | 0.98 (0.86-1.11) | 0.7 | 0.85 (0.6-1.2) | 0.36 | 1 (0.93-1.07) | 9.70E-01 | 9.70E-01 |
| *RAVER1* | 0.69 (0.53-0.9) | 0.0067 | 1.68 (1.42-2) | 1.20E-09 | 0.89 (0.68-1.16) | 0.4 | 0.94 (0.77-1.16) | 0.58 | 0.94 (0.67-1.33) | 0.75 | 1.09 (0.99-1.21) | 9.27E-02 | 1.87E-01 |
| *GCC1* | 0.92 (0.76-1.11) | 0.38 | 1.1 (0.97-1.25) | 1.30E-01 | 0.96 (0.79-1.16) | 0.65 | 0.98 (0.87-1.12) | 0.81 | 0.83 (0.59-1.18) | 0.3 | 1 (0.93-1.08) | 9.64E-01 | 9.70E-01 |
| *HSPB7* | 1 (0.83-1.21) | 1 | 1.02 (0.9-1.15) | 7.90E-01 | 0.84 (0.69-1.01) | 0.066 | 1.1 (0.97-1.25) | 0.15 | 0.87 (0.62-1.24) | 0.44 | 1 (0.94-1.08) | 8.92E-01 | 9.36E-01 |
| *SLC25A46* | 0.78 (0.6-1.03) | 0.077 | 0.62 (0.53-0.73) | 1.30E-08 | 0.74 (0.57-0.97) | 0.03 | 0.96 (0.78-1.18) | 0.69 | 0.8 (0.57-1.13) | 0.21 | 0.75 (0.67-0.83) | 1.93E-08 | **2.96E-07** |
| *KCNMB1* | 0.93 (0.77-1.13) | 0.48 | 1.06 (0.94-1.21) | 3.40E-01 | 0.89 (0.74-1.08) | 0.24 | 0.99 (0.87-1.12) | 0.84 | 0.88 (0.62-1.25) | 0.48 | 0.98 (0.91-1.06) | 6.24E-01 | 7.17E-01 |
| *CCDC97* | 0.78 (0.6-1.02) | 0.072 | 0.75 (0.64-0.89) | 7.00E-04 | 0.98 (0.75-1.28) | 0.89 | 0.91 (0.75-1.12) | 0.37 | 1.41 (1-2) | 0.049 | 0.87 (0.79-0.97) | 8.73E-03 | **3.01E-02** |
| *LRRC41* | 0.94 (0.78-1.13) | 0.5 | 0.68 (0.6-0.78) | 4.10E-09 | 0.8 (0.66-0.97) | 0.023 | 0.96 (0.85-1.1) | 0.58 | 1.48 (1.04-2.1) | 0.029 | 0.85 (0.79-0.92) | 4.37E-05 | **2.46E-04** |
| *TMEM44* | 1.02 (0.78-1.33) | 0.91 | 1.43 (1.21-1.69) | 2.30E-05 | 1.12 (0.85-1.46) | 0.42 | 1.2 (0.98-1.46) | 0.083 | 1.52 (1.07-2.15) | 0.019 | 1.26 (1.14-1.4) | 5.98E-06 | **3.76E-05** |
| *UBTD2* | 1.12 (0.86-1.47) | 0.39 | 0.7 (0.59-0.83) | 2.50E-05 | 1.12 (0.86-1.46) | 0.41 | 1.31 (1.07-1.61) | 0.009 | 1.59 (1.12-2.26) | 0.0083 | 1.01 (0.91-1.12) | 8.47E-01 | 8.97E-01 |
| *ZNF777* | 1.24 (0.95-1.63) | 0.11 | 1.26 (1.07-1.49) | 5.50E-03 | 0.97 (0.74-1.27) | 0.83 | 1.13 (0.93-1.39) | 0.22 | 1 (0.71-1.42) | 0.99 | 1.15 (1.04-1.28) | 6.29E-03 | **2.24E-02** |

NA: Not available in the datasets.

* 109 protein-coding mRNAs are included while the non-coding RNAs are not included in the microarray.

^#^ The dataset of breast cancer protein is not validated because the number of variables is low.

### Table S5. Study characteristics of the pan-cancer datasets

| Cancer type | Cancer full name | N_RNA-Seq_^#^ | N_miRNA-Seq_^#^ | N_final_^#^ | Median survival time (months) | Death proportion (%) |
| --- | --- | --- | --- | --- | --- | --- |
| ACC | Adrenocortical Carcinoma | 79 | 80 | 79 | 79.0 | 35.4 |
| BLCA | Bladder Urothelial Carcinoma | 408 | 409 | 405 | 34.0 | 43.7 |
| BRCA | Breast Invasive Carcinoma | 1090 | 1077 | 1071 | 129.5 | 13.7 |
| CESC | Cervical Squamous Cell Carcinoma and Endocervical Adenocarcinoma | 304 | 307 | 304 | 101.7 | 23.4 |
| CHOL | Cholangiocarcinoma | 36 | 36 | 36 | 40.1 | 50.0 |
| COAD | Colon Adenocarcinoma | 454 | 442 | 439 | 83.2 | 23.0 |
| DLBC | Lymphoid Neoplasm Diffuse Large B-cell Lymphoma | 48 | 47 | 47 | 211.1 | 19.1 |
| ESCA | Esophageal Carcinoma | 161 | 184 | 161 | 31.2 | 39.8 |
| HNSC | Head and Neck Squamous Cell Carcinoma | 500 | 523 | 495 | 54.9 | 43.4 |
| KICH | Kidney Chromophobe | 65 | 66 | 65 | 133.4* | 15.4 |
| KIRC | Kidney Renal Clear Cell Carcinoma | 530 | 516 | 512 | 90.8 | 33.0 |
| KIRP | Kidney Renal Papillary Cell Carcinoma | 288 | 291 | 288 | 135.1* | 15.3 |
| LAML | Acute Myeloid Leukemia | 151 | 103 | 85 | 17.0 | 65.9 |
| LGG | Brain Lower Grade Glioma | 510 | 511 | 506 | 79.9 | 24.5 |
| LIHC | Liver Hepatocellular Carcinoma | 371 | 372 | 367 | 55.7 | 34.6 |
| LUAD | Lung Adenocarcinoma | 513 | 513 | 507 | 49.2 | 36.5 |
| LUSC | Lung Squamous Cell Carcinoma | 501 | 478 | 475 | 56.3 | 42.1 |
| MESO | Mesothelioma | 86 | 87 | 86 | 18.5 | 84.9 |
| OV | Ovarian Serous Cystadenocarcinoma | 374 | 486 | 371 | 44.5 | 61.5 |
| PAAD | Pancreatic Adenocarcinoma | 177 | 178 | 177 | 19.9 | 52.0 |
| PCPG | Pheochromocytoma and Paraganglioma | 178 | 179 | 178 | 266* | 3.4 |
| PRAD | Prostate Adenocarcinoma | 495 | 494 | 491 | 145.2* | 2.0 |
| READ | Rectum Adenocarcinoma | 165 | 160 | 159 | 86.9* | 15.1 |
| SARC | Sarcoma | 259 | 259 | 257 | 65.4 | 37.7 |
| SKCM | Skin Cutaneous Melanoma | 468 | 448 | 447 | 79.5 | 46.3 |
| STAD | Stomach Adenocarcinoma | 375 | 436 | 372 | 28.9 | 39.2 |
| TGCT | Testicular Germ Cell Tumors | 134 | 134 | 134 | 234.6* | 3.0 |
| THCA | Thyroid Carcinoma | 502 | 506 | 501 | 165.3* | 3.2 |
| THYM | Thymoma | 119 | 124 | 119 | 131.0* | 7.6 |
| UCEC | Uterine Corpus Endometrial Carcinoma | 543 | 538 | 534 | 148.6* | 16.5 |
| UCS | Uterine Carcinosarcoma | 56 | 57 | 56 | 26.6 | 60.7 |
| UVM | Uveal Melanoma | 80 | 80 | 80 | 45.9 | 28.8 |

^#^ N_RNA-Seq_: Sample size with available RNA-Seq data; N_miRNA-Seq_: Sample size with available miRNA-Seq data; N_final_: Sample size with available RNA-Seq and miRNA-Seq data.

* Median survival time (MST) is not calculatable. We use mean survival time instead.

### Table S6. Sample size of tumor-normal pairs in each cancer type.

| Cancer type | N_RNA-Seq_^#^ | N_miRNA-Seq_^#^ | N_final_^#^ |
| --- | --- | --- | --- |
| BLCA | 19 | 19 | 19 |
| BRCA | 112 | 103 | 102 |
| CESC | 3 | 3 | 3 |
| CHOL | 9 | 9 | 9 |
| COAD | 41 | 8 | 8 |
| ESCA | 8 | 13 | 8 |
| HNSC | 43 | 43 | 42 |
| KICH | 23 | 25 | 23 |
| KIRC | 72 | 71 | 71 |
| KIRP | 31 | 34 | 31 |
| LIHC | 50 | 49 | 49 |
| LUAD | 57 | 46 | 19 |
| LUSC | 49 | 45 | 38 |
| PAAD | 4 | 4 | 4 |
| PCPG | 3 | 3 | 3 |
| PRAD | 52 | 52 | 52 |
| READ | 9 | 3 | 2 |
| SARC | 2 | 0 | 0 |
| SKCM | 1 | 2 | 1 |
| STAD | 27 | 41 | 27 |
| THCA | 58 | 59 | 58 |
| THYM | 2 | 2 | 2 |
| UCEC | 23 | 21 | 21 |

^#^ N_RNA-Seq_: Sample size with available RNA-Seq data; N_miRNA-Seq_: Sample size with available miRNA-Seq data; N_final_: Sample size with available RNA-Seq and miRNA-Seq data.

### Table S7. Gene list used to generate the m6A subtypes in each cancer

| Cancer type | Gene list |
| --- | --- |
| ACC | ALL^#^ |
| BLCA | ZMYM1,LAMTOR5,HDGF,ID2,CXCR4,CDCP1,IGF2BP2,let-7g,miR-143,miR-145,SRF,MYB,FSCN1,IGF2BP3,HNRNPA2B1,miR-25,miR-106b,miR-671,MYC,YAP1,FOXM1,METTL3,ASB2,miR-1266,RARA,TK1,miR-21,miR-744,BCL2,CEBPA,AXL,E2F1,UBE2C,YTHDF1,P2RX6,miR-33a |
| BRCA | HDGF,CXCR4,ITGA6,PDCD1,miR-375,CDCP1,SOX2,IGF2BP2,LEF1,ADAM19,miR-143,miR-145,SRF,MYB,IGF2BP3,EGFR,miR-29a,miR-29b-1,miR-671,KIAA1429,MYC,miR-126,GATA3-AS1,GATA3,YAP1,miR-125b-1,FOXM1,WIF1,SOCS2,ARHGAP5-AS1,miR-1266,FTO,RARA,IGF2BP1,TK1,miR-21,CEBPA,IL11,E2F1,KCNK15-AS1,UBE2C,P2RX6,miR-33a,XIST |
| CESC | ALL |
| CHOL | YTHDF2,ZMYM1,SRSF11,RBM15,LAMTOR5,HDGF,GAS5-AS1,GAS5,ID2,CXCR4,ITGA6,CTNNB1,RBM15B,IGF2BP2,DANCR,YTHDC1,LEF1,YTHDC2,miR-143,SRSF3,SRF,WTAP,FSCN1,HNRNPA2B1,miR-25,miR-106b,IKBKB,YTHDF3,KIAA1429,NOTCH1,miR-126,EIF3A,BNIP3,MALAT1,RELA,YAP1,miR-125b-1,FOXM1,SP1,GLI1,HNRNPC,METTL3,ARHGAP5-AS1,HIF1A,USP7,FTO,PHLPP2,METTL16,TP53,ALKBH5,RARA,SP2,TK1,miR-21,KEAP1,BRD4,CEBPA,MZF1,E2F1,SRSF6,UBE2C,SNAI1,YTHDF1,RBMX,TAZ,miR-221,miR-222 |
| COAD | GAS5-AS1,GAS5,ITGA6,miR-375,CTNNB1,let-7g,DANCR,LEF1,ADAM19,miR-143,miR-145,FSCN1,IGF2BP3,EGFR,miR-106b,miR-29b-1,miR-671,MYC,NOTCH1,miR-126,BNIP3,miR-125b-1,FOXM1,WIF1,SOCS2,METTL3,ASB2,miR-1266,PHLPP2,TP53,TK1,miR-21,miR-744,BCL2,KEAP1,AXL,IL11,MZF1,E2F1,SRSF6,UBE2C,SNAI1,YTHDF1,RBMX,TAZ,miR-221 |
| DLBC | ALL |
| ESCA | HDGF,HNRNPA2B1,miR-126,FOXM1,HNRNPC,TK1,miR-21,BRD4,E2F1,UBE2C,YTHDF1,miR-221 |
| HNSC | ZMYM1,HDGF,ID2,ITGA6,miR-375,SOX2,IGF2BP2,LEF1,ADAM19,miR-143,miR-145,FSCN1,IGF2BP3,EGFR,miR-29a,miR-25,miR-106b,miR-29b-1,miR-671,KIAA1429,miR-126,BNIP3,LINC00958,ETS1,miR-125b-1,FOXM1,GLI1,WIF1,HIF1A,ASB2,PHLPP2,IGF2BP1,TK1,miR-21,miR-744,BRD4,AXL,IL11,E2F1,UBE2C,SNAI1,YTHDF1,P2RX6,miR-221 |
| KICH | ZMYM1,SRSF11,LAMTOR5,HDGF,ID2,ITGA6,CTNNB1,CDCP1,SEC62,DANCR,YTHDC1,LEF1,miR-143,miR-145,SRSF3,SRF,WTAP,miR-29a,miR-106b,miR-29b-1,miR-671,NOTCH1,GATA3-AS1,GATA3,PTEN,BNIP3,NEAT1,MALAT1,YAP1,ETS1,FOXM1,NANOG,GLI1,SOCS2,HNRNPC,HIF1A,ASB2,miR-1266,USP7,FTO,PHLPP2,RARA,SP2,IGF2BP1,TK1,miR-744,BCL2,IL11,E2F1,SRSF6,UBE2C,SNAI1,XIST,miR-221,miR-222 |
| KIRC | GAS5,ID2,CXCR4,PDCD1,IGF2BP2,DANCR,LEF1,ADAM19,FSCN1,IGF2BP3,EGFR,miR-25,miR-106b,miR-671,MYC,NOTCH1,miR-126,GATA3-AS1,GATA3,BNIP3,ETS1,FOXM1,WIF1,SOCS2,ARHGAP5-AS1,HIF1A,ASB2,FTO,RARA,TK1,miR-21,BCL2,CEBPA,AXL,IL11,E2F1,KCNK15-AS1,UBE2C,P2RX6,miR-33a,TAZ |
| KIRP | CXCR4,IGF2BP2,DANCR,miR-143,miR-145,MYB,IGF2BP3,EGFR,miR-25,miR-106b,miR-671,MYC,miR-126,GATA3-AS1,GATA3,ETS1,FOXM1,SP1,SOCS2,miR-1266,PHLPP2,TP53,TK1,miR-21,miR-744,CEBPA,AXL,IL11,E2F1,KCNK15-AS1,UBE2C,miR-33a,TAZ,miR-222 |
| LAML | ALL |
| LGG | ALL |
| LIHC | ZMYM1,SRSF11,LAMTOR5,HDGF,GAS5-AS1,GAS5,ID2,ITGA6,PDCD1,CTNNB1,RBM15B,SOX2,IGF2BP2,DANCR,LEF1,miR-145,FSCN1,IGF2BP3,HNRNPA2B1,miR-25,miR-106b,IKBKB,YTHDF3,KIAA1429,NOTCH1,EIF3A,NEAT1,MALAT1,RELA,YAP1,miR-125b-1,FOXM1,SP1,GLI1,SOCS2,HNRNPC,METTL3,ARHGAP5-AS1,HIF1A,miR-1266,USP7,PHLPP2,METTL16,TP53,RARA,SP2,IGF2BP1,TK1,miR-21,BCL2,KEAP1,BRD4,CEBPA,AXL,MZF1,E2F1,UBE2C,YTHDF1,RBMX,TAZ,miR-221,miR-222 |
| LUAD | ZMYM1,HDGF,GAS5,ID2,PDCD1,CDCP1,SOX2,IGF2BP2,DANCR,LEF1,ADAM19,miR-143,miR-145,SRF,FSCN1,IGF2BP3,EGFR,miR-29b-1,NOTCH1,BNIP3,ETS1,miR-125b-1,FOXM1,WIF1,SOCS2,HNRNPC,HIF1A,TP53,IGF2BP1,TK1,miR-21,CEBPA,IL11,E2F1,UBE2C,YTHDF1,P2RX6,miR-33a |
| LUSC | ZMYM1,HDGF,ID2,ITGA6,CDCP1,SOX2,IGF2BP2,DANCR,LEF1,ADAM19,miR-143,miR-145,MYB,FSCN1,IGF2BP3,HNRNPA2B1,EGFR,miR-29a,miR-106b,miR-671,KIAA1429,MYC,miR-126,BNIP3,LINC00958,ETS1,FOXM1,WIF1,SOCS2,HNRNPC,ARHGAP5-AS1,HIF1A,USP7,PHLPP2,TP53,RARA,IGF2BP1,TK1,miR-21,miR-744,BCL2,KEAP1,BRD4,AXL,IL11,E2F1,KCNK15-AS1,UBE2C,SNAI1,YTHDF1,miR-33a,miR-222 |
| MESO | ALL |
| OV | ALL |
| PAAD | ALL |
| PCPG | ALL |
| PRAD | GAS5,ID2,miR-375,SOX2,IGF2BP2,DANCR,LEF1,ADAM19,miR-143,MYB,miR-25,miR-106b,miR-671,MYC,NOTCH1,miR-126,GATA3-AS1,GATA3,LINC00958,YAP1,FOXM1,WIF1,ASB2,miR-1266,TK1,miR-21,miR-744,BCL2,AXL,P2RX6,miR-221 |
| READ | SRSF11,HDGF,LEF1,MYC,NOTCH1,miR-125b-1,METTL3,ASB2,PHLPP2,miR-21,miR-744,BCL2,E2F1,UBE2C,SNAI1,YTHDF1,RBMX,TAZ |
| SARC | ALL |
| SKCM | ALL |
| STAD | ZMYM1,SRSF11,RBM15,HDGF,ITGA6,CTNNB1,CDCP1,IGF2BP2,LEF1,miR-143,miR-145,SRF,MYB,FSCN1,IGF2BP3,HNRNPA2B1,miR-25,miR-106b,miR-671,NOTCH1,GATA3-AS1,GATA3,BNIP3,ETS1,FOXM1,GLI1,WIF1,SOCS2,ARHGAP5-AS1,ASB2,miR-1266,USP7,TP53,IGF2BP1,TK1,miR-21,miR-744,BCL2,IL11,E2F1,SRSF6,UBE2C,SNAI1,YTHDF1,TAZ,miR-221,miR-222 |
| TGCT | ALL |
| THCA | ZMYM1,GAS5,ITGA6,miR-375,IGF2BP2,DANCR,miR-143,miR-145,SRF,MYC,miR-126,LINC00958,GLI1,PHLPP2,RARA,TK1,miR-21,BCL2,CEBPA,E2F1,SNAI1,miR-221,miR-222 |
| THYM | ALL |
| UCEC | YTHDF2,RBM15,LAMTOR5,HDGF,CDCP1,SEC62,let-7g,DANCR,LEF1,METTL14,miR-143,miR-145,SRF,IGF2BP3,HNRNPA2B1,EGFR,miR-29a,miR-25,miR-106b,miR-671,MYC,PTEN,BNIP3,LINC00958,ALKBH3,YAP1,ETS1,FOXM1,GLI1,SOCS2,HIF1A,ASB2,miR-1266,FTO,PHLPP2,TP53,TK1,miR-21,miR-744,BCL2,AXL,IL11,E2F1,UBE2C,P2RX6,miR-33a,miR-221 |
| UCS | ALL |
| UVM | ALL |

^#^ALL: For those cancer types with no data available from normal tissues, all the genes above were used to calculate the m6A subtypes


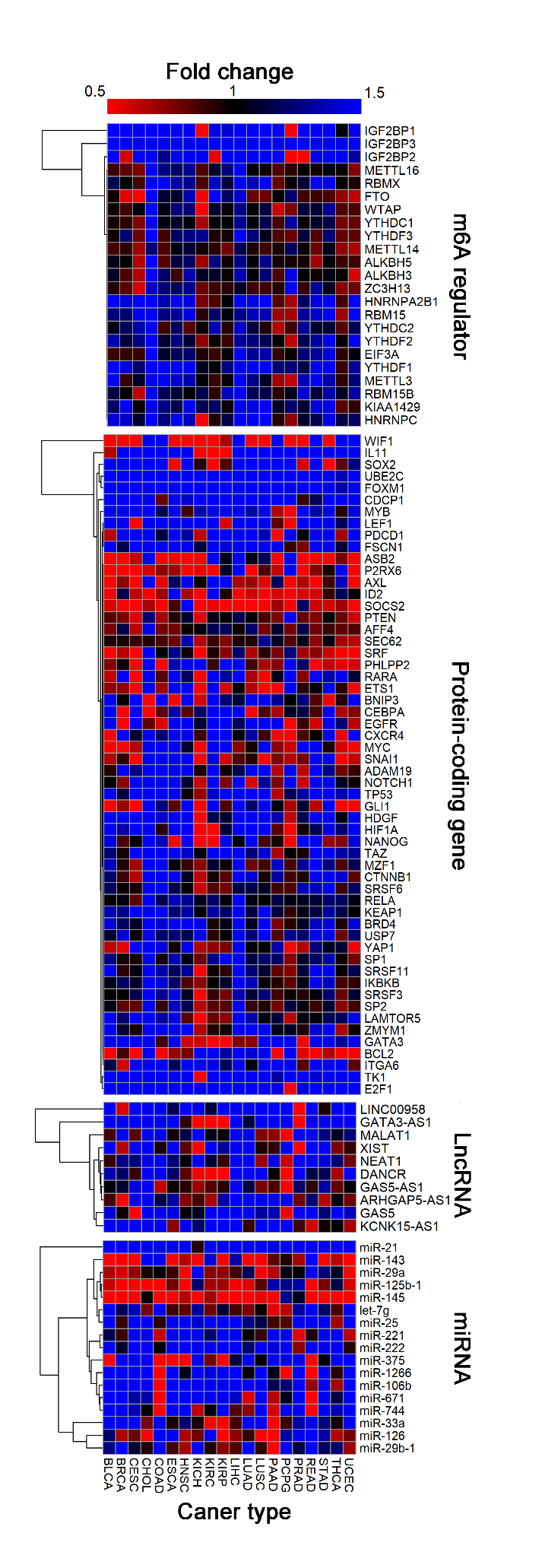


### Figure S1. Heatmap of the fold change (FC) values in pan-cancer.


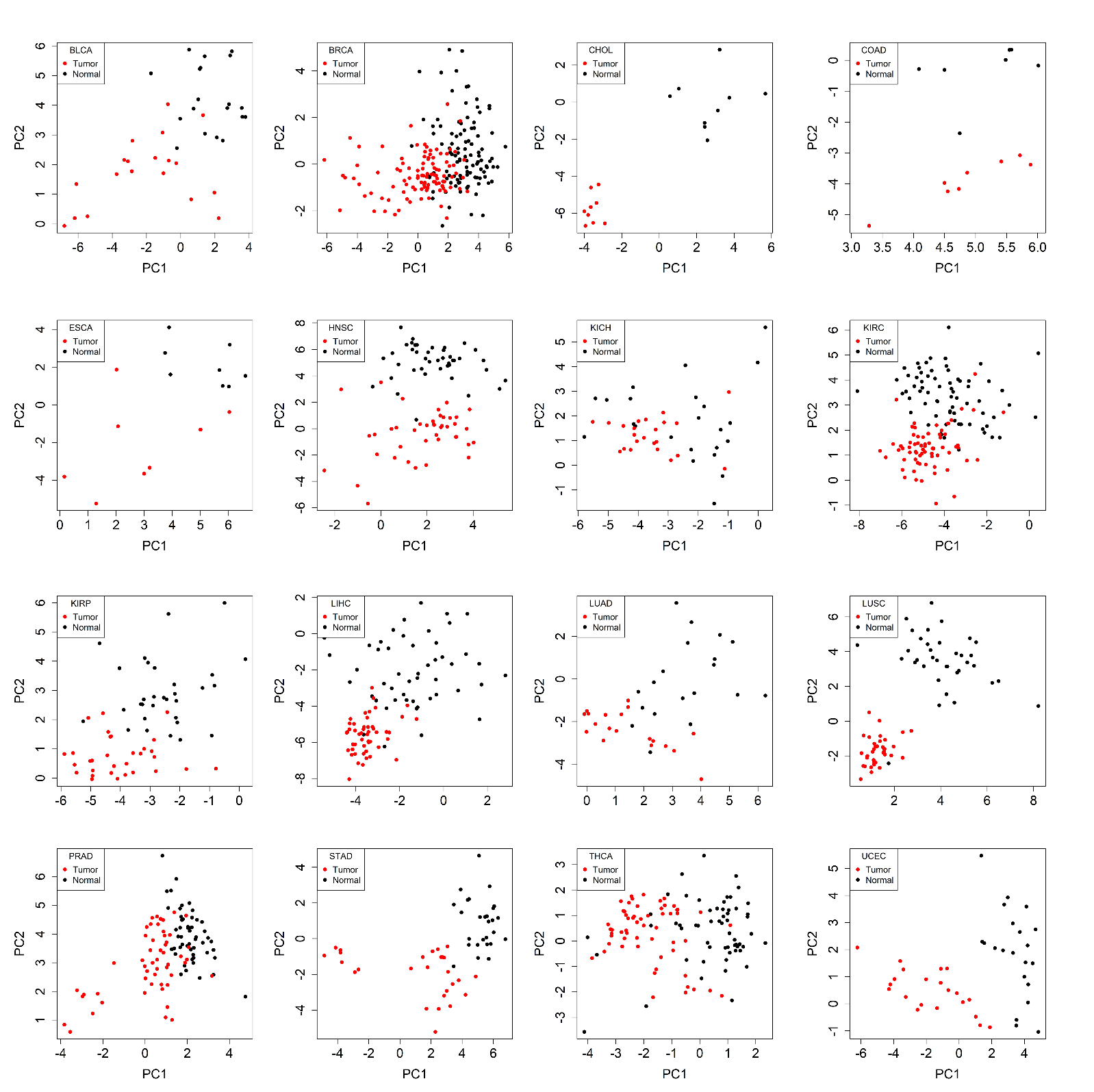


### Figure S2. PCA plots of m6A related genes in tumors and adjacent normal tissues


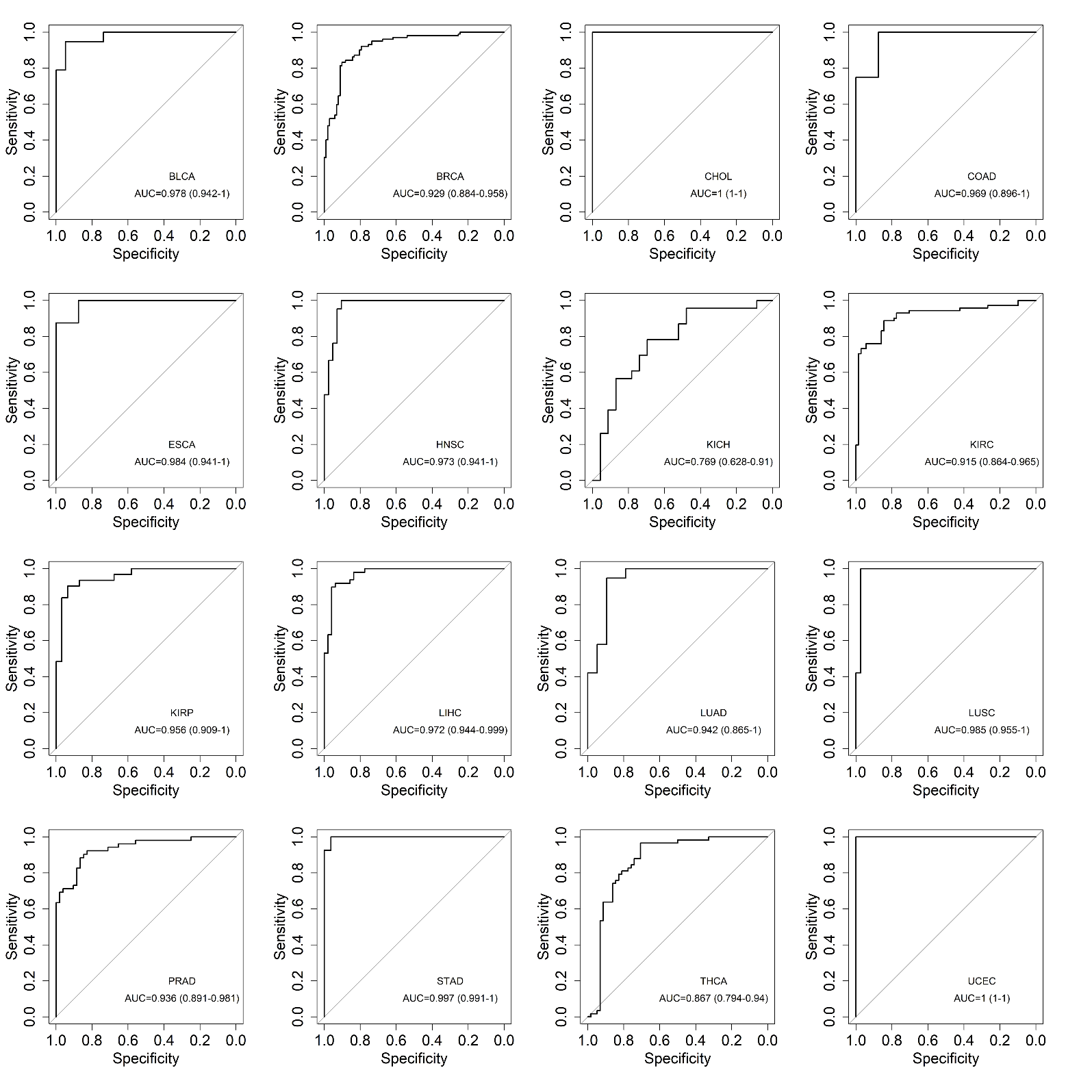


### Figure S3. Receiver-operating characteristic curves of predicted performance to discriminate from tumor and normal tissues


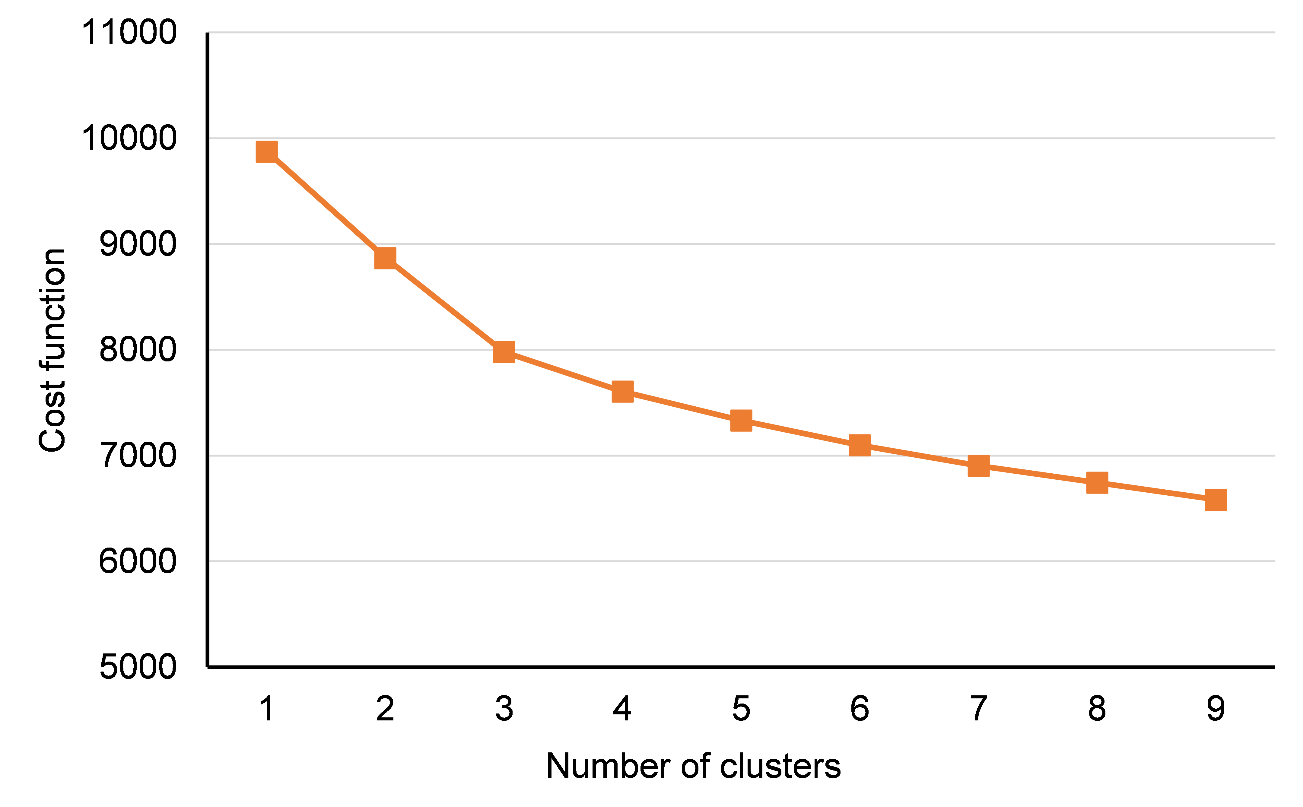


### Figure S4. Average cost of different clusters using the Elbow method.

The cost function is calculated as the average values of 32 cancer types to keep the number of clusters unified.


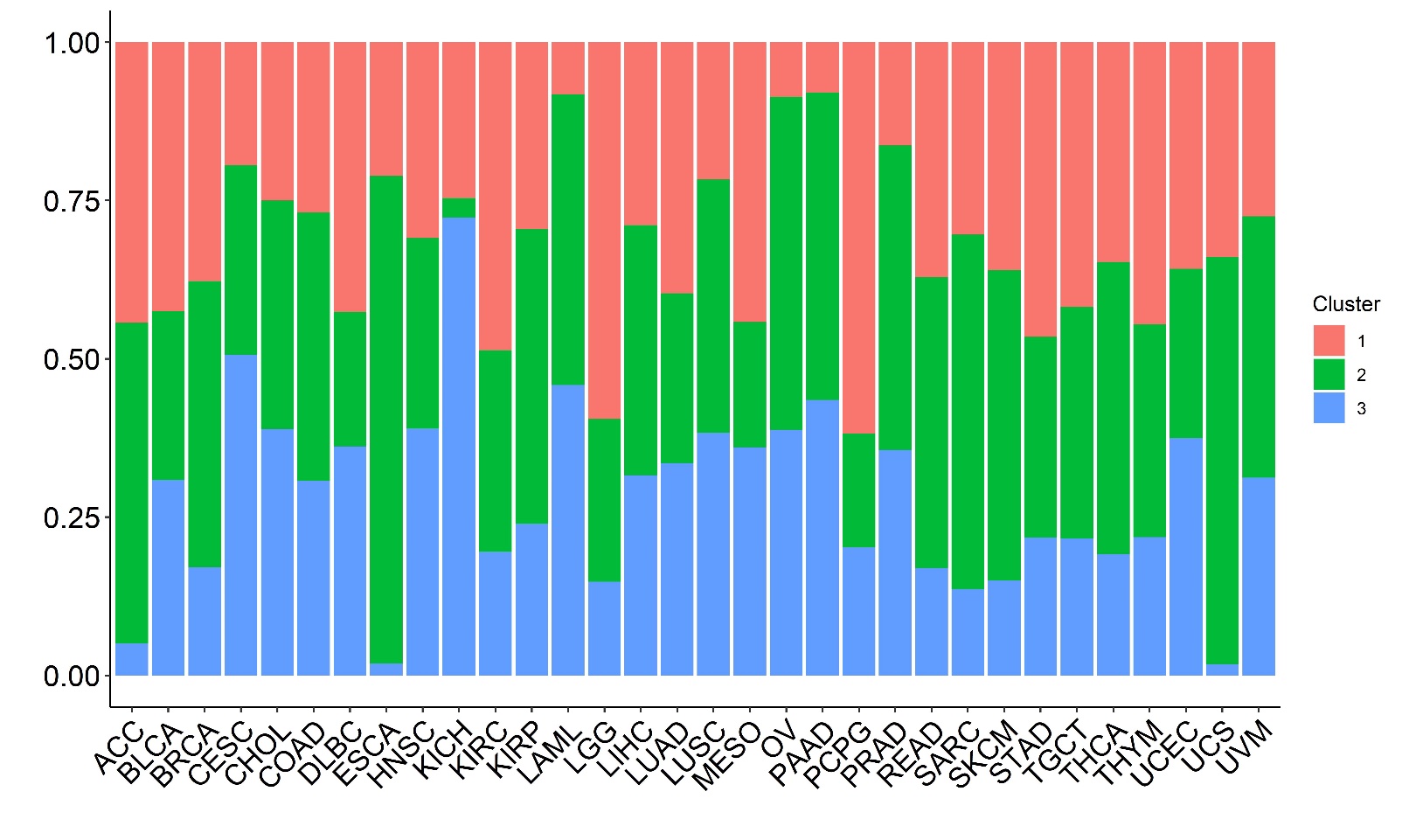


Proportion

### Figure S5. Distribution of the m6A subtype across each cancer type


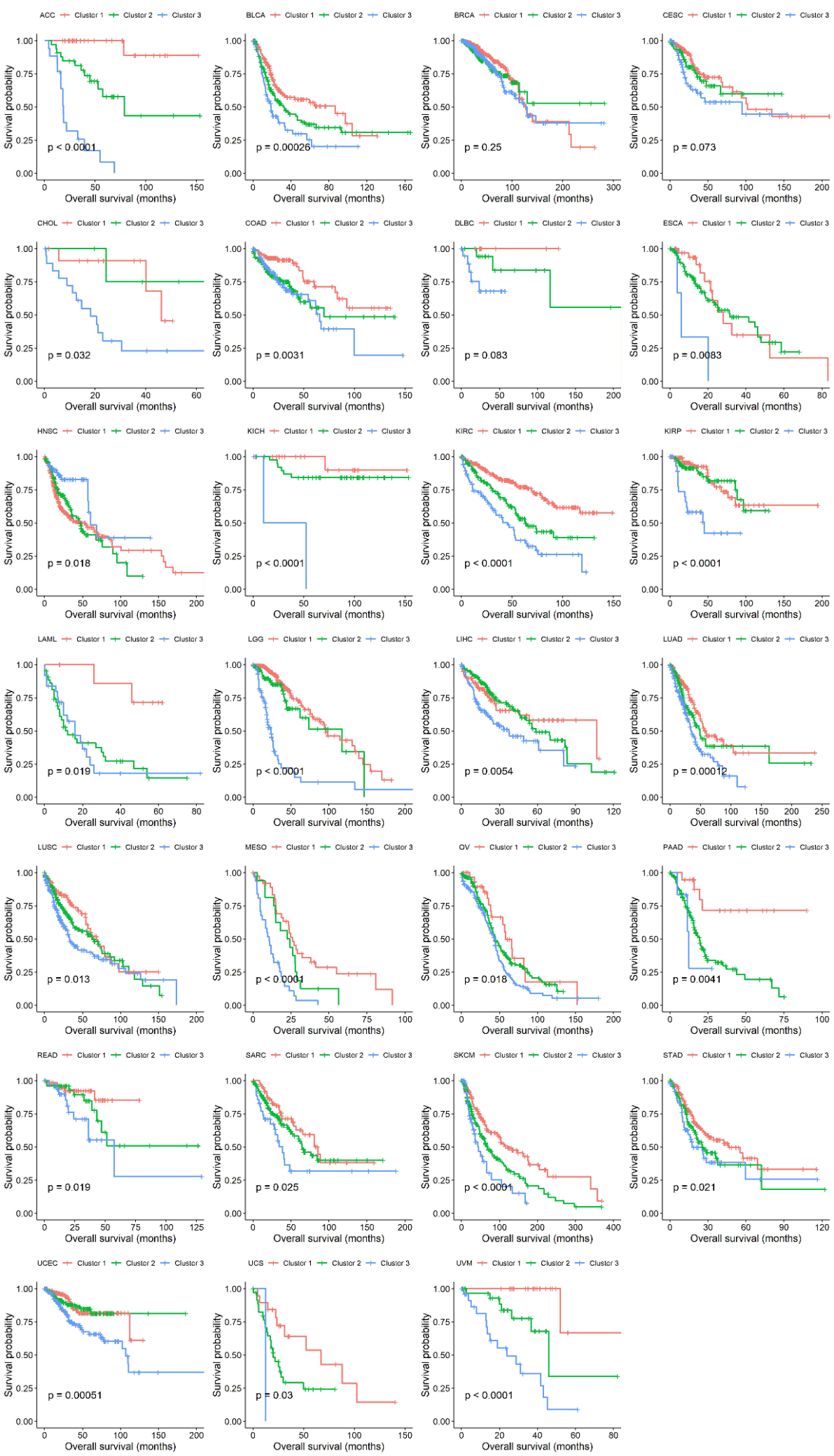


### Figure S6. Kaplan-Meier plots of m6A subtypes and overall survival in pan-cancer


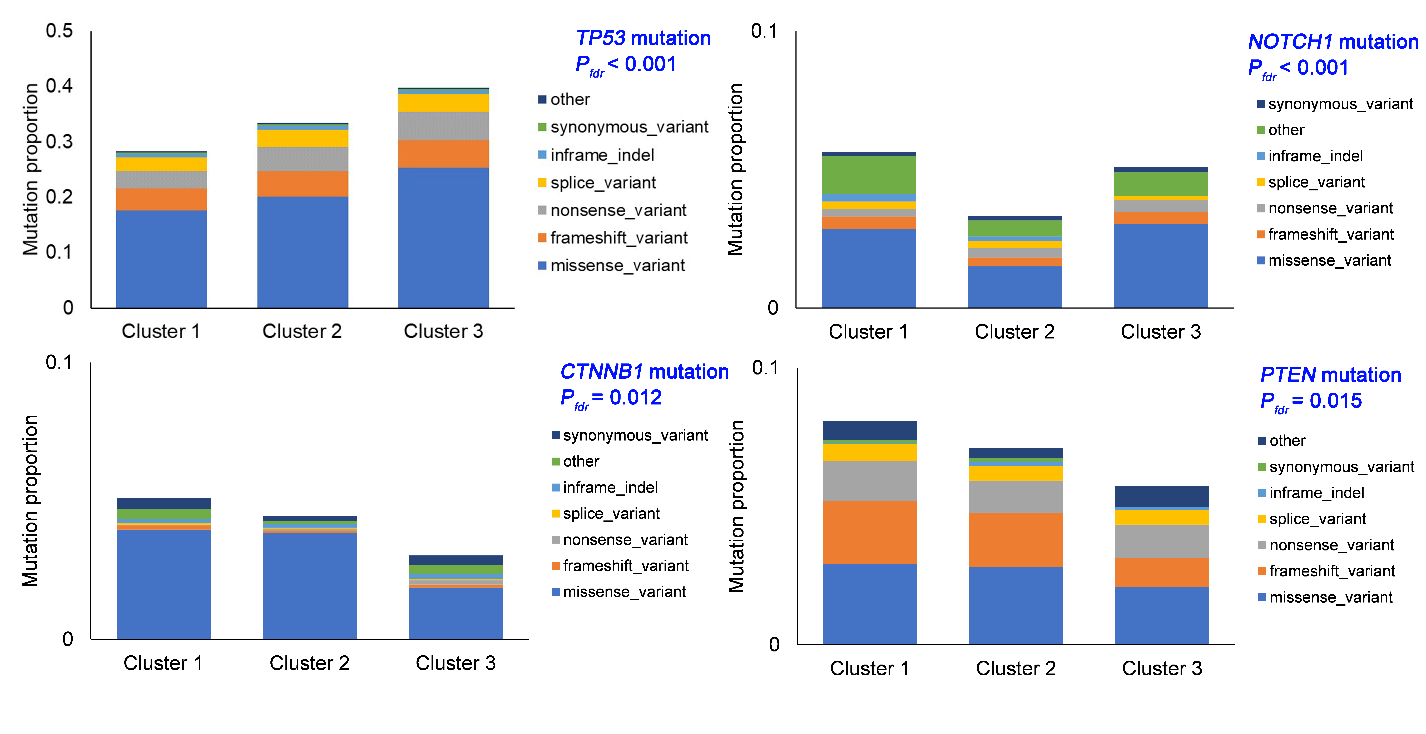


### Figure S7. Associations between somatic mutations and m6A subtypes.

Distributions of four somatic mutations were significantly different among different m6A subtypes (FDR-correct *P* values of Chi-square test <0.05), including *TP53, NOTCH1, CTNNB1*, and *PTEN.*


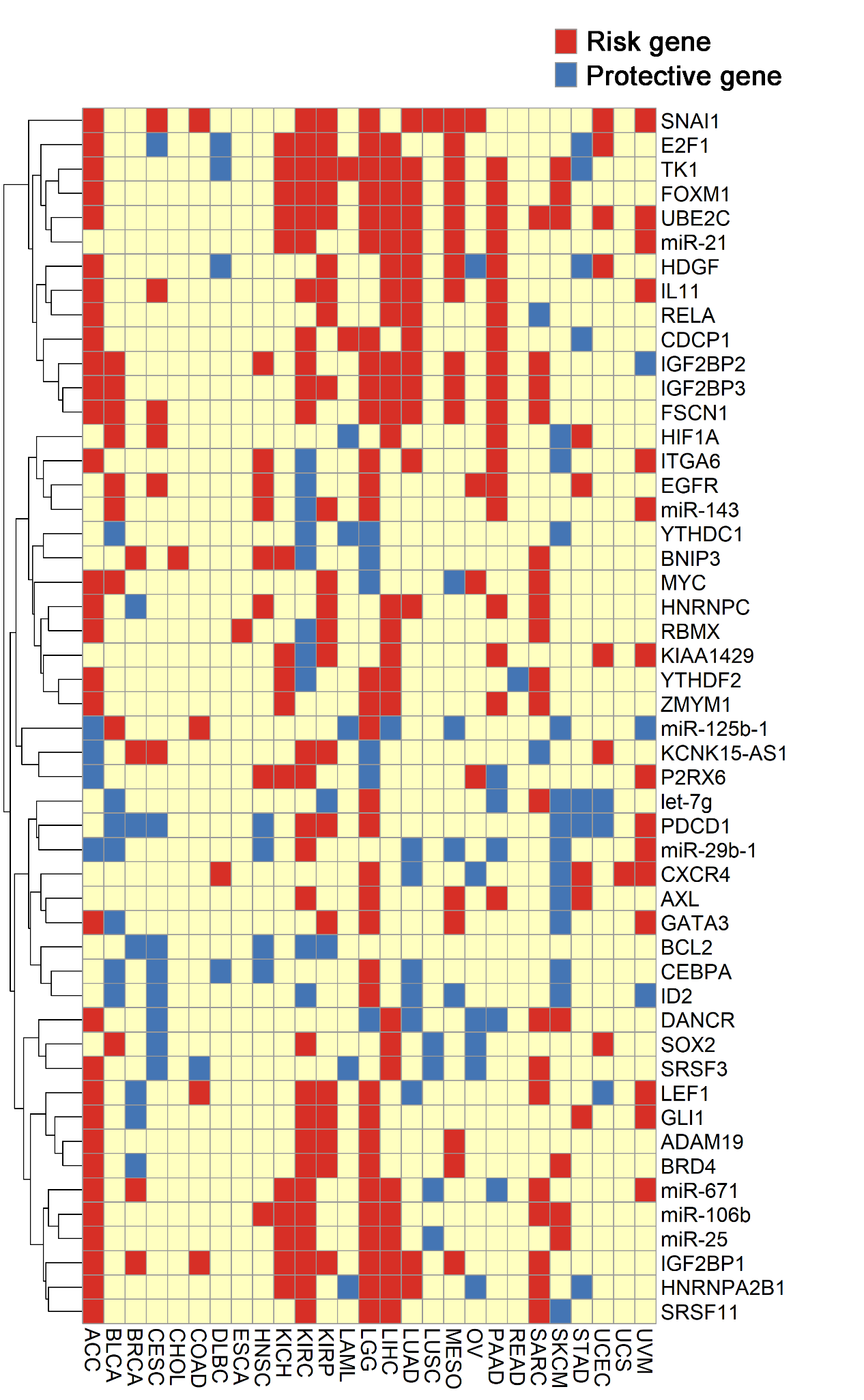


### Figure S8. Heatmap of the genes associated with overall survival in at least five cancer types and used to generate an m6A signature


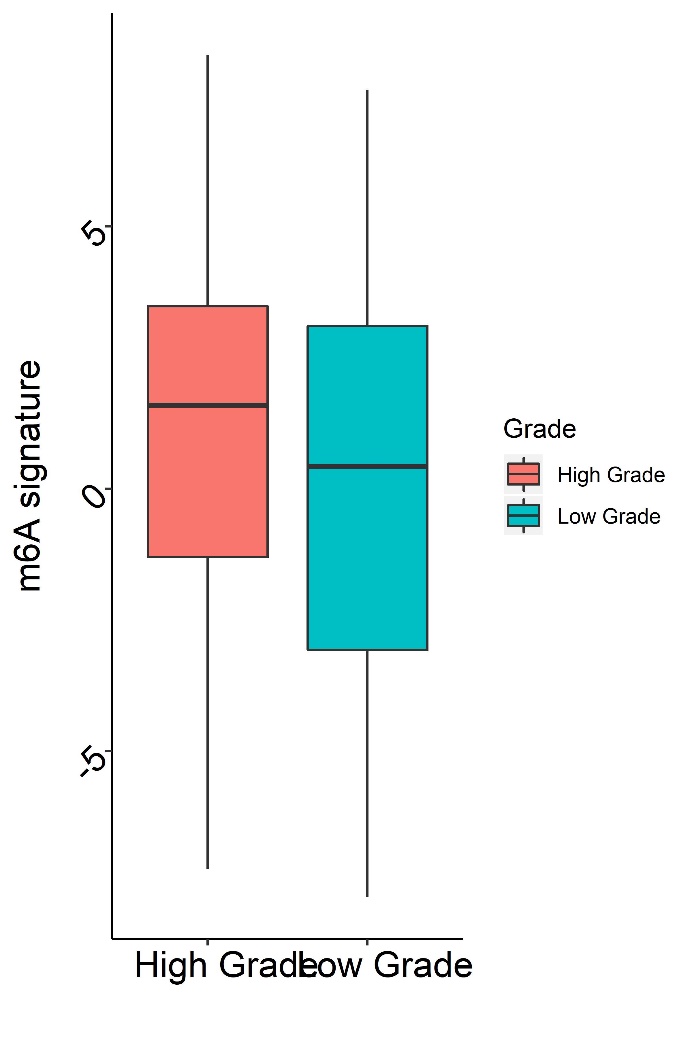


### Figure S9. Distribution of m6A signature in low-grade (grade 1&2) and high-grade (grade 3&4) patients


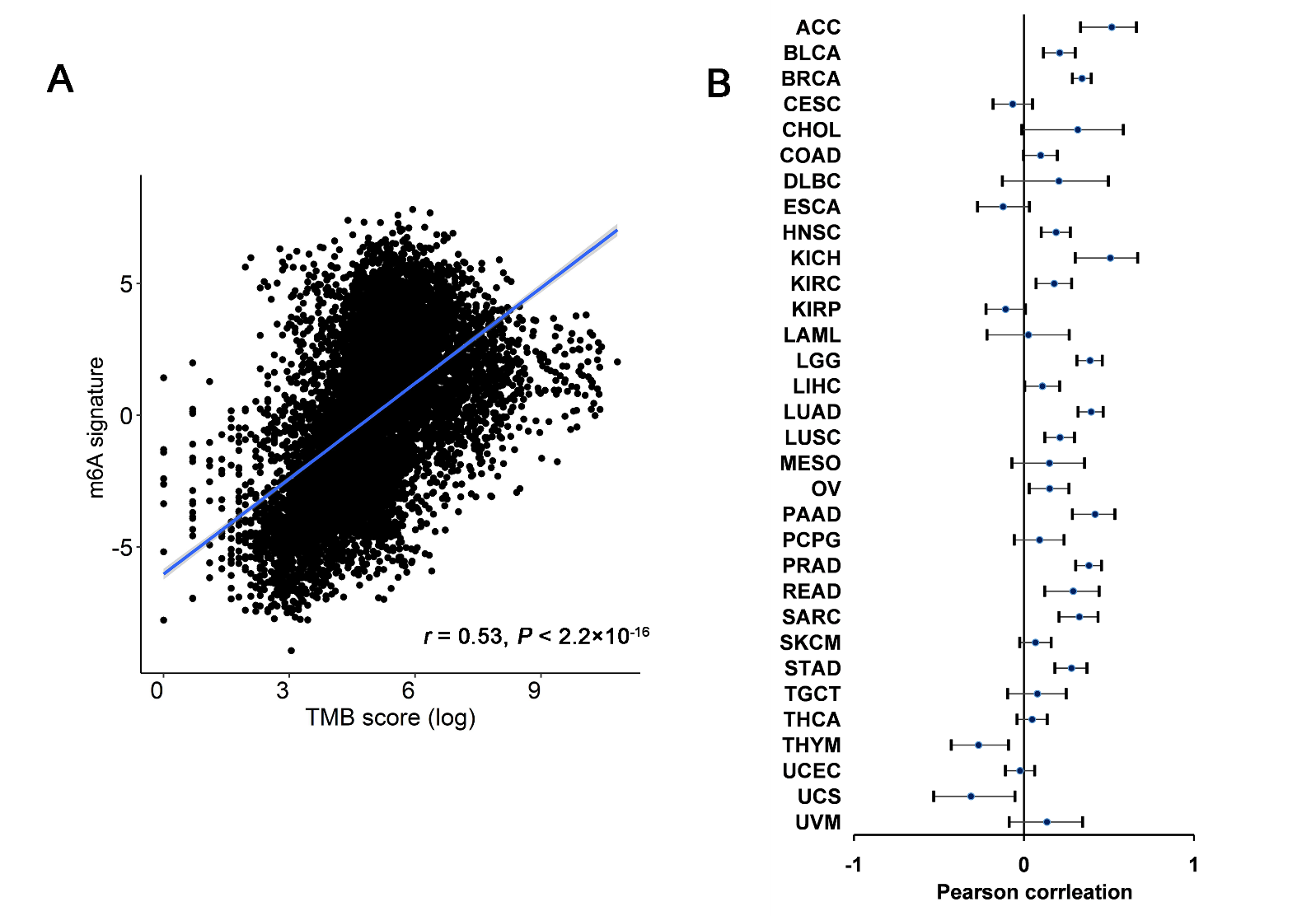


### Figure S10. Associations between tumor mutation burden score and m6A signature in pan-cancer


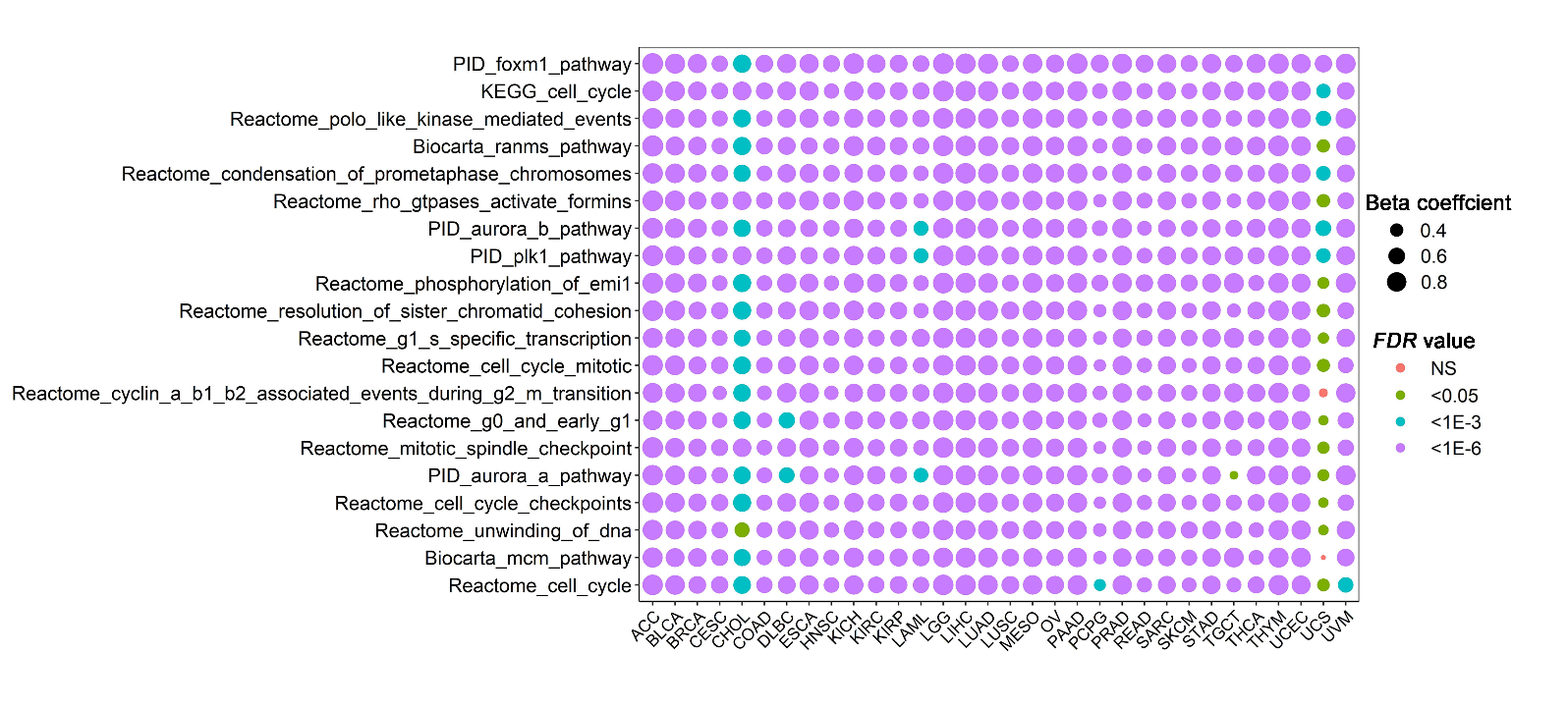


### Figure S11. The top 20 biological pathways that are associated with the m6A signature.

The size of the dot represents the effect size in the multivariable linear model. The color of the dot represents the false discovery rate (FDR) value levels in the model.


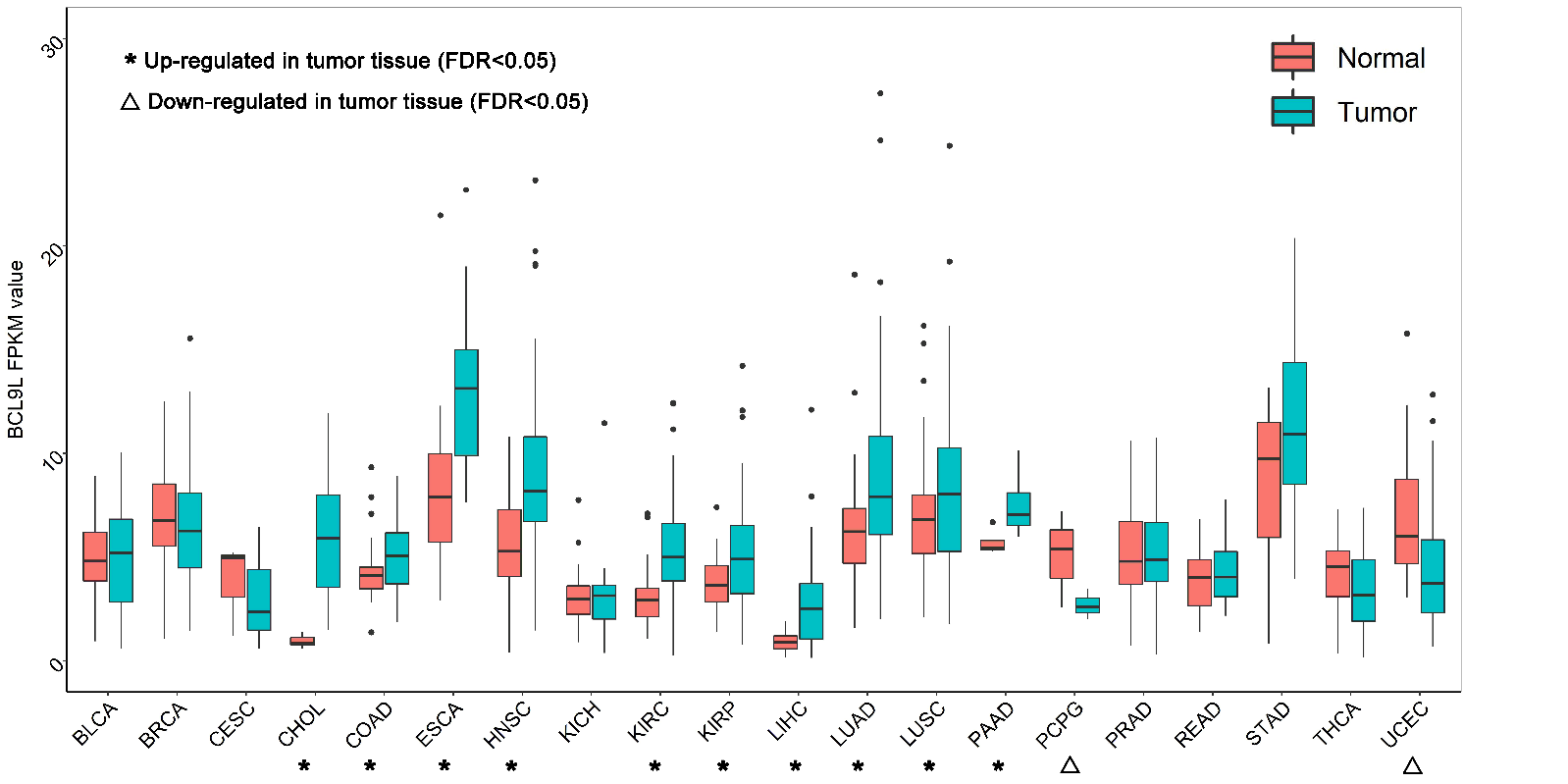


### Figure S12. Differential comparison of *BCL9L* in TCGA tumor and adjacent-normal tissues


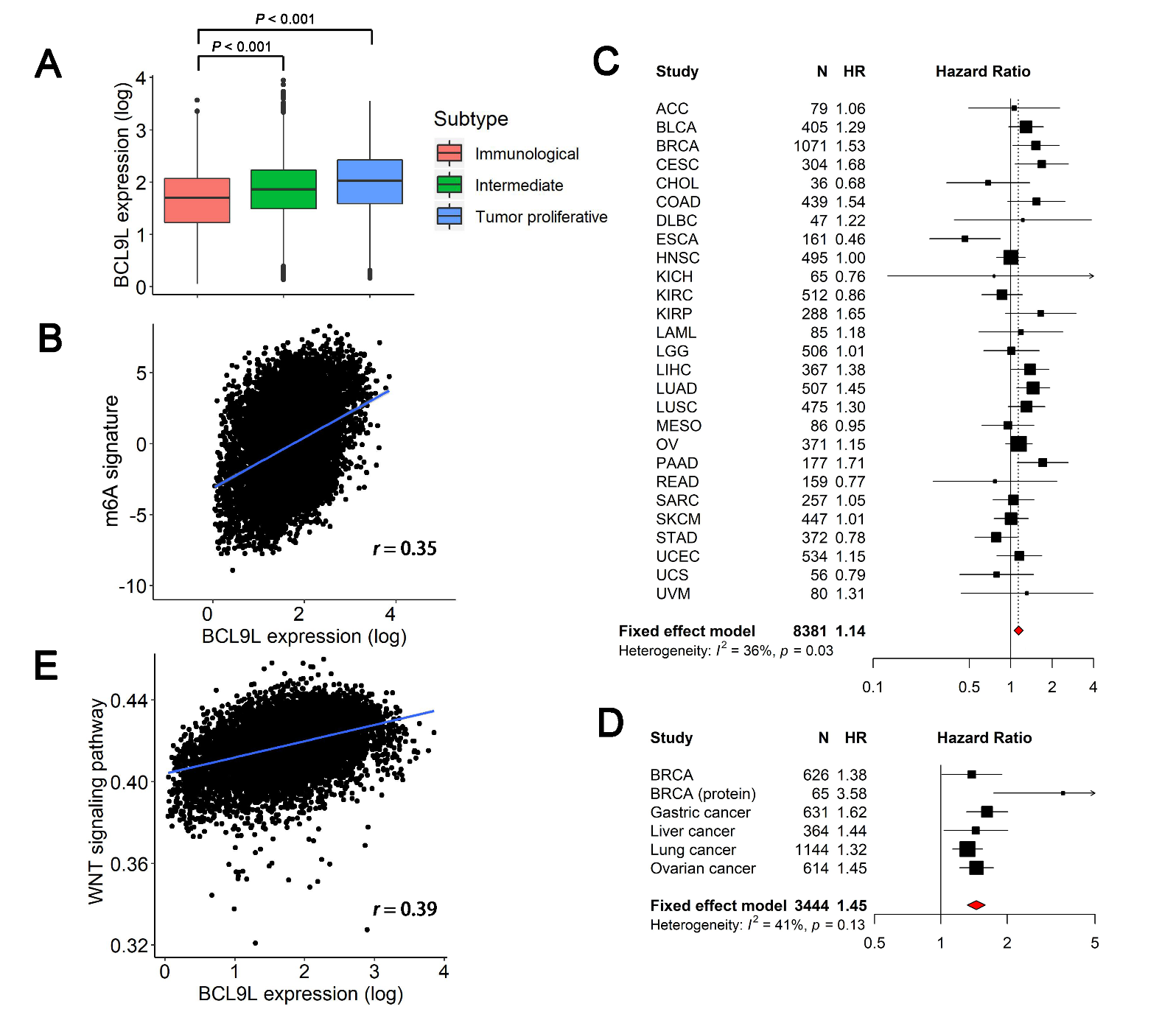


### Figure S13. BCL9L and survival outcome in pan-cancer

(A) Distribution of *BCL9L* expression in different m6A subtypes. (B) Correlation between *BCL9L* expression and m6A signature. (C) Meta-analysis of the associations of *BCL9L* expression and overall survival in TCGA pan-cancer. (D) External independent validation of the prognostic value of *BCL9L* in GEO datasets. (E) Correlation between *BCL9L* expression and the ssGSEA score of the Wnt signaling pathway.


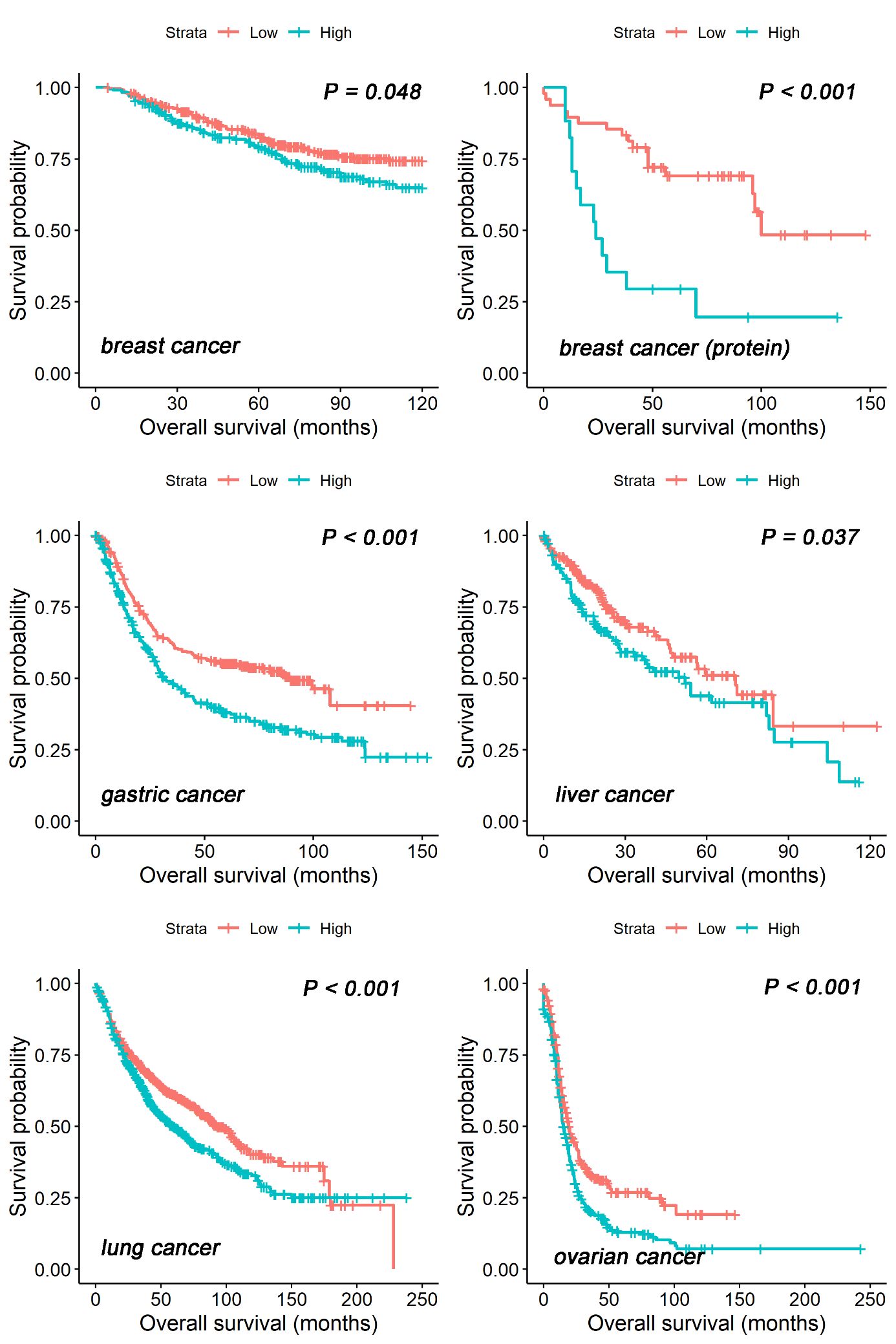


### Figure S14. External validation of *BCL9L* prognostic value in public GEO datasets


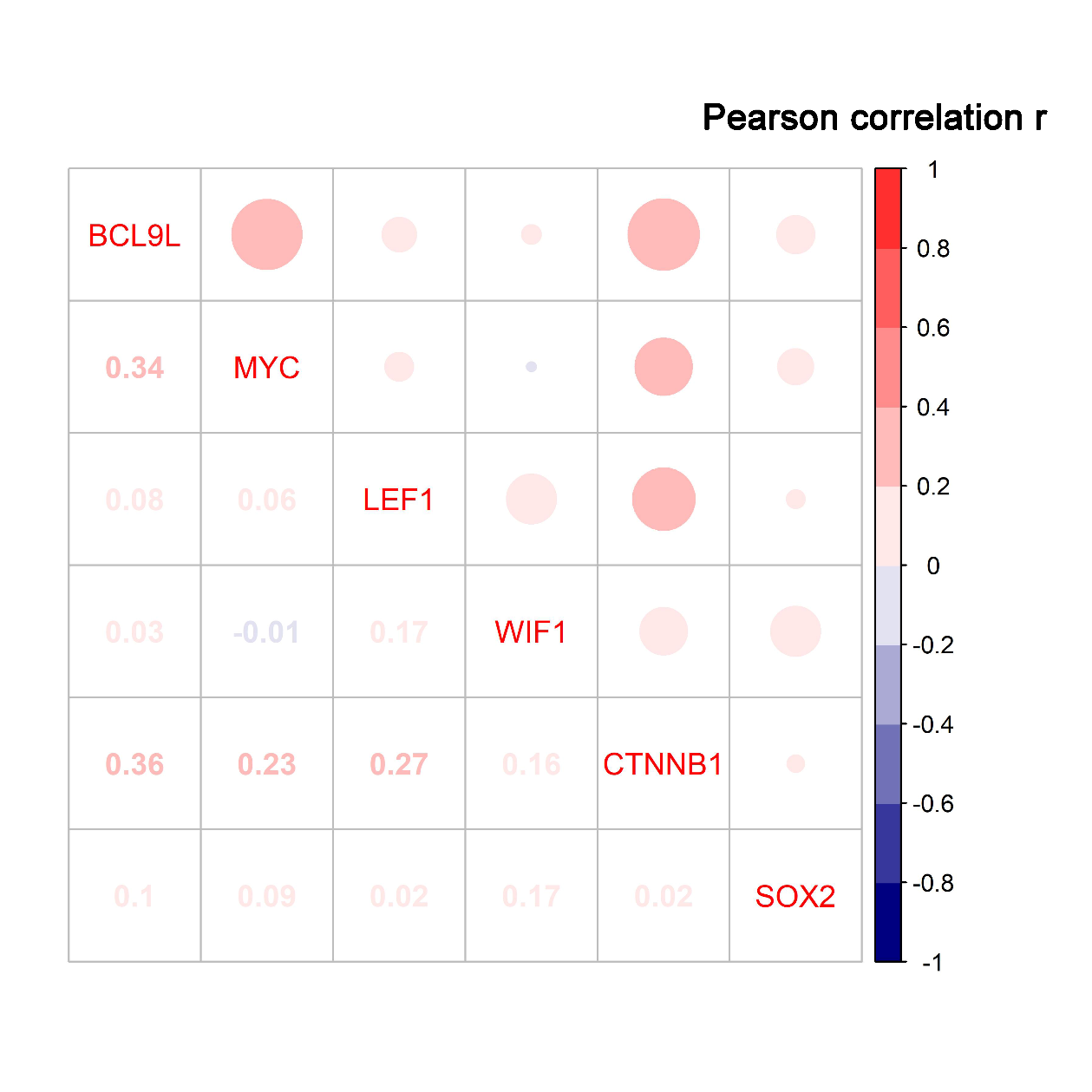


### Figure S15. Correlations between *BCL9L* and m6A interactive genes in the Wnt signaling pathway
